# Distribution and genome structures of temperate phages in acetic acid bacteria

**DOI:** 10.1038/s41598-021-00998-w

**Published:** 2021-11-03

**Authors:** Koki Omata, Naruhiro Hibi, Shigeru Nakano, Shuji Komoto, Kazuki Sato, Kei Nunokawa, Shoichi Amano, Kenji Ueda, Hideaki Takano

**Affiliations:** 1 Life Science Research Center, College of Bioresource Sciences, Nihon University, Kanagawa, Japan; 2 Mizkan Holdings Co., Ltd, Aichi, Japan

**Keywords:** Microbiology, Applied microbiology, Bacteriophages, Microbial genetics

## Abstract

Acetic acid bacteria (AAB) are industrial microorganisms used for vinegar fermentation. Herein, we investigated the distribution and genome structures of mitomycin C-inducible temperate phages in AAB. Transmission electron microscopy analysis revealed phage-like particles in 15 out of a total 177 acetic acid bacterial strains, all of which showed morphology similar to myoviridae-type phage. The complete genome sequences of the six phages derived from three strains each of *Acetobacter* and *Komagataeibacter* strains were determined, harboring a genome size ranging from 34,100 to 53,798 bp. A phage AP1 from *A. pasteurianus* NBRC 109446 was predicted as an active phage based on the genomic information, and actually had the ability to infect its phiAP1-cured strain. The attachment sites for phiAP1 were located in the 3’-end region of the *tRNA*^*ser*^ gene. We also developed a chromosome-integrative vector, p2096int, based on the integrase function of phiAP1, and it was successfully integrated into the attachment site of the phiAP1-cured strain, which may be used as a valuable tool for the genetic engineering. Overall, this study showed the distribution of mitomycin C-inducible temperate phages in AAB, and identified the active temperate phage o f *A. pasteurianus*.

## Introduction

Acetic acid bacteria (AAB) are Gram-negative and obligate aerobic bacteria belonging to the order *Rhodospirillales* of the class *alphaproteobacteria*^1–3^. AAB are frequently isolated from sugar-containing or acidic materials such as fermented food, beverages, animal organs, fruits, flowers, insect guts, and soil^1–3^. The AAB strains belonging to the *Acetobacter*, *Gluconobacter*, and *Komagataeibacter* genera have been mainly used to brew vinegar and produce functional molecules^1^. *A. pasteurianus* is used to brew cereal vinegars by liquid-state or solid-state fermentation in Asia countries such as Japan and China. One of the remarkable abilities of AAB is the oxidization of ethanol, sugar, or sugar alcohol, under the presence of oxygen, to organic acids such as acetic acid and gluconic acid^1^. This specific ability has been used for industrial fermentation of vinegar, a highly important organic acid in the food industry that contributing to food, bioengineering and medical fields.

Bacteriophages (phages) are bacteria-infective viruses with great diversity and are the most abundant biological entities^4^; they play an important role in bacterial ecology and evolution, including gene reservoir and horizontal transfer of genetic materials^5–7^. Phages are divided into four life cycles including the lytic cycle, the lysogenic cycle, pseudolysogeny, and chronic infection or shedding^8^. Lytic (also called virulent) phages amplify their genome after infection, and lyse the host cells to infect other healthy host cells. Temperate (also called lysogenic) phages have two modes: prophages integrate into host chromosome and propagate with host genome until the host is active, while prophages are induced (transfer to lytic phase) when physiological and chemical stress are generated by the treatment of UV irradiation, mytomycin C (MMC), and reactive oxygen species (ROS) as a DNA-damaging reagent^9^. The temperate phage-derived integrase gene encodes a member of the tyrosine or serine family recombinase catalyzing a site-specific recombination between two specific sites: the attachment sites of bacterial host (*attB*) and phage (*attP*)^10^. The lytic and temperate phages comprise a majority of the life cycle of phages.

Morphology and genome structures of virulent and temperate phages have been well studied in industrial bacteria, while a few studies on AABs phage have been reported as described below. As virulent phages of AAB, phages GW6210 and JW2040 isolated from rotten apples showed virulence to *Gluconobacter oxydans* ATCC 621 and *G. oxydans* VP1 204 JW^11^, respectively. As temperate phages, phage Acm1 from *Acetobacter methanolicus* MB5814^12^, phage A-1 from *G. oxydans* strain E^13^, and more recently, a novel tectivirus phage GC1 infecting *G. cerinus* associated with wine-making^14^ were reported. To the best of our knowledge, there have been no reports on phages associated with a major vinegar-producing *A. pasteurianus* and a cellulose-producing *K. xylinum*.

Phage-like particles in vinegar fermentation have been experimentally observed in the culture broth when fermentation is halted (our unpublished observation). However, little has been characterized on virulent and temperate phages of AABs as described above. Here, we performed a comprehensive investigation of AAB temperate phages induced by MMC treatment. Understanding the phage diversity and genomic structures will aid in improving biotechnological processes for preventing the fermentation from halting. In this study, we detected 15 myoviridae-type temperate phages among a total of 177 AAB strains, and we determined the complete genome sequences of the six temperate phages produced from *Acetobacter* and *Komagataibacter* spp. We also report that a phage AP1 (designated as phiAP1) from *A. pasteurianus* NBRC 109446 has the ability to infect its phiAP1-cured strain. Finally, we have developed a chromosome-integrative vector based on the function of phiAP1 as a valuable tool for genetic engineering.

## Results

### Distribution of phage-like elements in the genomes of acetic acid bacteria (AAB)

Viral DNAs of temperate phages are ubiquitously found in the genomes of phylogenetically diverse Gram-positive and Gram-negative bacteria; however, little is known about AAB temperate phages compared to other industrially used bacteria. We first searched for prophage-like elements using a web-based tool PHAge Search Tool (PHAST 2016.12.23 Updated version; http://phast.wishartlab.com/)^15^ against 22 whole genome sequences derived from the genera *Acetobacter*, *Gluconobacter*, *Gluconacetobacter*, and *Komagataeibacter*. As listed in Table S1, the harbor of phage-like elements in these genomes were predicted in all of the genome-sequenced AABs, and a total of 94 prophage-like elements were found in the 22 AAB strains. The average number of temperate phages per acetic acid bacterium is 4.3, which is higher than that of other bacteria, 2.6^16^, indicating that AAB strains potentially have a larger number of prophage-like elements than other bacteria.

The PHAST-based prediction grouped the phages into three types: intact (active phage), incomplete (degenerate phage), and questionable (where it was impossible to predict whether the phage is active or degenerate). The temperate phages predicted as active were found in 12 of the 22 AAB strains. To the best of our knowledge, there are no previous reports of temperate phages derived from *A. pasteurianus and K. xylinum*; however, the PHAST analysis suggests that temperate prophage-like elements might be widespread in these bacterial groups. These results strongly suggest the existence of active temperate phages in these industrial microorganisms.

### Screening of lysogens in AAB

To survey the distribution of active temperate phages existing as prophage within the AAB genomes, we first screened lysogenic AAB strains mainly belonging to the *Acetobacter*, *Gluconobacter*, *Gluconoacetobacter*, and *Komagataeibacter* genera, which were obtained from publically or commercially available culture collections such as JCM, NBRC, IAM, ATCC and DSMZ. A total of 177 AAB strains (Table S2) were grown on YPG solid media in the absence or presence of low concentration of mitomycin C (MMC), which is frequently used as an inducer agent for temperate phage due to its inhibitory activity of DNA synthesis. As listed in Table S3, 27 out of 177 strains showed a MMC-sensitive growth profile, in which the growth inhibition by MMC might be due to host cells lysis followed by phage induction. This growth inhibition was also confirmed when strains were grown in MMC-containing liquid shaking culture. The above 27 strains were composed of 10 strains of *Acetobacter* spp, seven strains of *Komagataeibacter* spp, one strain of *Gluconoacetobacter* sp, six strains of *Gluconobacter* spp, and one strain each of *Frateuria* sp., *Acidomonas methanolica* ATCC 43582, and *Ameyamaea chiangmaiensis* NBRC 103196. These results imply that approximately 15.2% of the AAB strains retain a MMC-inducible temperate phage.

### Transmission electron microscopy (TEM) analyses of phage particles

**Figure 1:**
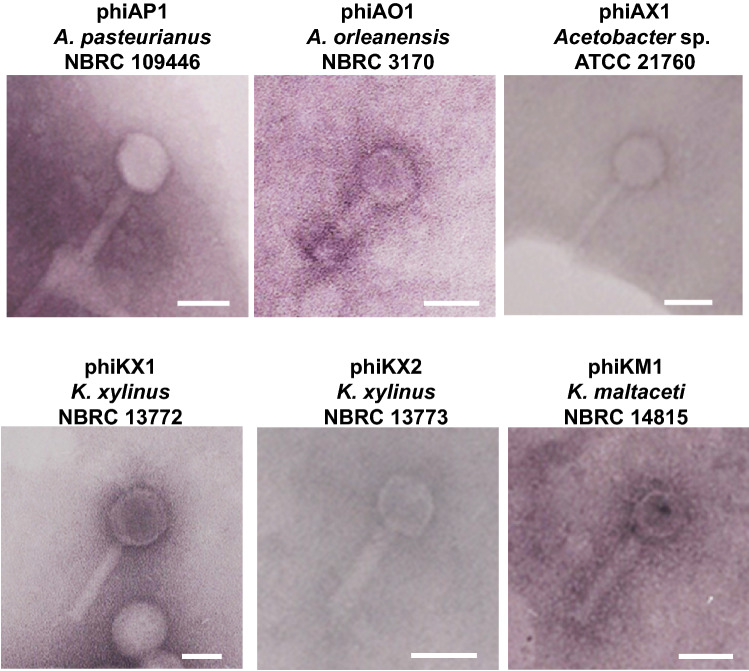
TEM photographs of temperate phages produced by acetic acid bacteria. Phage particles of acetic acid bacteria (AAB) were photographed with TEM. Fractions were prepared from AAB grown in YPG-1% glucose medium containing mitomycin C (MMC) with shaking culture for 48 h. Bars, 50 nm.

To confirm whether the above 27 strains actually produced phage particles in response to MMC, we performed TEM analyses using the phage-containing fractions, which were prepared by concentrating the supernatant from the culture broth of the MMC-treated lysogens (See Materials and Methods). We successfully observed phage particles in 15 out of 27 lysogens with TEM analyses (Table 1, Fig. 1, and Fig. S1). The number of the lysogens observed was four for *Acetobacter* strains, six for *Komagataeibacter*, three for *Gluconobacter*, one for *Acidomonas*, and one for *Ameyamaea*. TEM analyses also revealed features of their morphology; the observed phage particles retained their heads and contractile tails. Based on morphology, all of the phages were classified into the myoviridae family with icosahedral capsids 41 ± 5 nm in diameter, and tails 156 ± 6 nm in length (Table 1). Overall, these results suggest that a large proportion (8.4%) of AAB are capable of producing myoviridae-type temperate phages. The MMC induction of phage particles indicates that the trigger is linked to the SOS response of the bacterial host. The growth inhibition observed in other MMC-sensitive strains might due to the generation of degenerate phage.

**Table 1:** Morphological characteristics of AAB temperate phages.

Host strain	Isolation source^a^	Head^b^	Tail^b^	n^c^	Tail-fiber^d^
***Acetobacter***					
*A. orleanensis* NBRC 3170	Manufacture of vinegar	43.1 (0.0)	46.6 (1.4)	3	-
*A. orleanensis* ATCC 6438	Manufacture of vinegar	45.0 (1.3)	83.3 (13.6)	3	-
*A. pasteurianus* NBRC 109446	Vinegar fermentor	68.8 (0.0)	86.1 (3.9)	3	-
*Acetobacter* sp. ATCC 21760	-	44.8 (0.8)	135.8 (9.5)	3	-
***Komagataeibacter***					
*K. maltaceti* NBRC 14815	Malt vinegar brewery acetifiers	47.4 (1.3)	104.6 (6.1)	3	-
*K. xylinus* NBRC 13693	-	52.1	60.3	1	-
*K. xylinus* NBRC 13772	Film in fermentor of vinegar	65.6 (1.5)	98.9 (3.0)	3	-
*K. xylinus* NBRC 13773	Film in fermentor of vinegar	55.0 (4.1)	95.0 (10.8)	3	-
*K. xylinus* ATCC 14851	Vinegar brew	60.2	82.6	1	-
*K. xylinus* ATCC 53264	-	62.1 (0.0)	56.0 (0.9)	2	-
***Gluconobacter***					
*G. cerinus* IAM 1832	Apple	50.0 (1.5)	94.6 (1.5)	3	-
*G. cerinus* ATCC 23775	Baker's yeast	54.5 (0.0)	83.3 (7.7)	3	-
*G. frateurii* IAM 1815	-	59.2 (2.2)	71.8 (2.2)	3	-
**Others**					
*Acidomonas methanolica* ATCC 43582	Ditch sludge	44.4 (0.0)	75.3 (1.7)	3	-
*Ameyamaea chiangmaiensis* NBRC 103196	Flower of red ginger, *Alpinia purpurea*	67.5 (0.0)	15.0 (0.0)	2	-

^a^Dashes indicate unknown; ^b^Values are Mean (SD) of phage size (nm); ^c^n is measurement number of phage particle; ^d^Dashes indicate not detected.

### Complete genome sequences of AAB temperate phages

**Figure 2:**
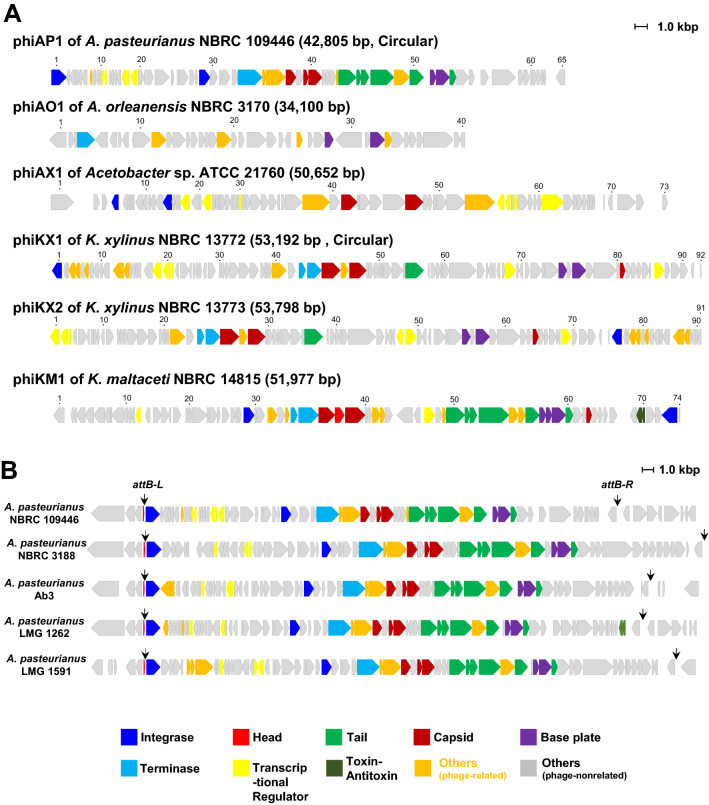
Schematic representation of temperate phage genomes in AAB. (**A**) Genome structure and gene organization of the temperate phages determined in this study. Number above arrow represent ORF number in each temperate phage (annotations for each gene product are shown in Table S4). Numbers in parentheses indicate the genome size and structure of the phages. (**B**) Distribution of phiAP1-like prophage elements in the genomes of *A. pasteurianus* strains. Downward arrowheads indicate the putative *attB-L* and *attB-R* sites.

To reveal the genome structures and gene composition of the temperate phages observed in the TEM analyses, we selected one or two strains from each genus/species among the lysogens isolated from the vinegar fermenter. We then sequenced the whole genomes of six temperate phages using next generation sequencing technique. Genomic DNAs were purified from the phage-fraction prepared by concentrating the supernatant of the MMC-treated AABs (See Materials and Methods). The complete genome sequences without any gaps were determined in the six phages, and each gene product was annotated. Table S4 shows the annotation of each phage genome. In each strain, one phage genome was detected in the genome-sequencing analysis. As shown in Fig. 2A, we designated the six phages as phiAP1 from *A. pasteurianus* NBRC 109446, phiAO1 from *A. orleanensis* NBRC 3170, phiAX1 from *Acetobacter* sp. ATCC 21760, phiKX1 from *Komagataeibacter xylinus* NBRC 13772, phiKX2 from *K. xylinus* NBRC 13773, and phiKM1 from *K. maltaceti* NBRC 14815.

Figure 2A shows the predicted gene organization based on phage genome information. Genome sizes range from 34,100 to 53,798 bp. Circular genome were confirmed in phiAP1 and phiKX1. The molecular G + C content of each genome is 52.4% for phiAP1, 61.4% for phiAO1, 56.0% for phiAX1, 60.2% for phiKX1, 60.0% for phiKX2, and 62.2% for phiKM1 (Table S4). The molecular G + C contents of *A. pasteurianus* IFO 3283–01 are 50. 7% in the chromosome and 55.8% in the six plasmids^17^, and that of *K. xylinus* E25 are 62.6% in the chromosome and 57. 8% in the five plasmids^18^. The G + C contents of the six phages lie in the between the values of chromosome and plasmids in both genera.

In each of the phage genomes, open reading frames (ORFs) were found: 65 in phiAP1, 40 in phiAO1, 72 in phiAX1, 91 in phiKX1, 90 in phiKX2, and 73 in phiKM1 (Fig. 2A and Table S4). Most genes are predicted to be composed of an operon structure, because the initiation codon and stop codon of translation are close or overlapped in the ORFs.

Phage integrase recognizes two sites, a bacterial attachment site (*attB*) and a phage attachment site (*attP*), and catalyzes the site-specific recombination between *attB* and *attP* to generate the integrated state of phage flanked with *attB-L* and *attB-R*^10^. Most integrase are classified into two families, tyrosine and serine recombinase families^10^. Integrase genes were found in all prophage genomes studied except for that of *A. orleanesis* NBRC 3170 (Fig. 2A). These integrases are classified into tyrosine family integrases based on Pfam search. The existence of integrase in the phage genome suggests that these phages serve as a temperate type.

To examine the functionality of phages, their genomes were analyzed by PHAST. The two phages, phiAP1 and phiKM1, were predicted as active phages. In contrast, phiAO1, phiAX1, and phiKX1 were predicted as incomplete, indicating degenerate prophages; and phiKX2 was predicted as questionable. This implies that the three phages predicted as incomplete have an ability to assemble the phage body structure, but lack the infection to the host strain. In support of this prediction, phiAP1 and phiKM1 retains many phage-related genes in their genomes (Fig. 2A). In contrast, phiAO1 harbors only nine phage-related genes out of a total 40 genes. Thus, these data support that phiAP1 and phiKM1 serve as functional phages, which are able to infect AAB strains.

### Distribution of phiAP1-like elements in *Acetobacter* spp

We searched the distribution of phiAP1-like elements in AABs using publically available genomic information. Four strains of *A. pasteurianus* including NBRC 3188, Ab3, LMG 1262, and LMG 1591 retained a phiAP1-like prophage element in their genomes (Fig. 2B). In contrast, *A. pasteurianus* NBRC 3283 and 386B have not phiAP1-like element in their genomes. The composition and direction of the phage-related genes located in the center and right region of the genome are highly conserved among the four *Acetobacter* strains, while the composition of the small ORFs located in the flanking region of *attB-L* are slightly different in each strain. We also found that putative *attB-L* and *attB-R* have a high similarity to each other among the four *Acetobacer* spp. as described below. PHAST analysis suggested that all of the above phiAP1-like phages are active, suggesting that they have the ability to infect *Acetobacter* spp. The high similarity of phiAP1-like elements among the five strains suggests that their origin is identical, and that they are spread by phage infection.

### Construction of a phiAP1-cured strain in *A. pasteurianus* NBRC 109446

Among the phages observed with TEM analyses, phiAP1 is predicted as an active temperate phage, and phiAP1-like elements are found in other *A. pasteurianus* strains as described above. Among the detected temperate phages, our study focused on phiAP1 derived from *A. pasteuianus* NBRC 109446, because the host strain was isolated from a vinegar fermentation facility in Japan. To examine the ability of phiAP1 in infecting *A. pasteurianus* NBRC 109446, we first constructed a phiAP1-cured strain, due to the general presence of a system preventing multiple phage infection which protects their lysogenic host by superinfection exclusion and homo-immunity against infecting phages^19^.

We analyzed approximately 1,200 colonies of MMC-treated *A. pasteurianus* NBRC 109446 using PCR analysis to confirm the loss of the phiAP1 prophage genome. Tween 80 was added to prevent phage reinfection by inhibiting phage adsorption^20^. As a result, we obtained a single phiAP1-cured strain among the ca. 1,200 colonies, which was designated as a C-27 strain. The inability of the C-27 strain to produce MMC-induced phages was confirmed with TEM analyses. This suggests that phiAP1 is the sole MMC-inducible phage in *A. pasteurianus* NBRC 109446 in the cultures condition used in this study.

### Attachment sites recognized by phiAP1 Integrase

**Figure 3:**
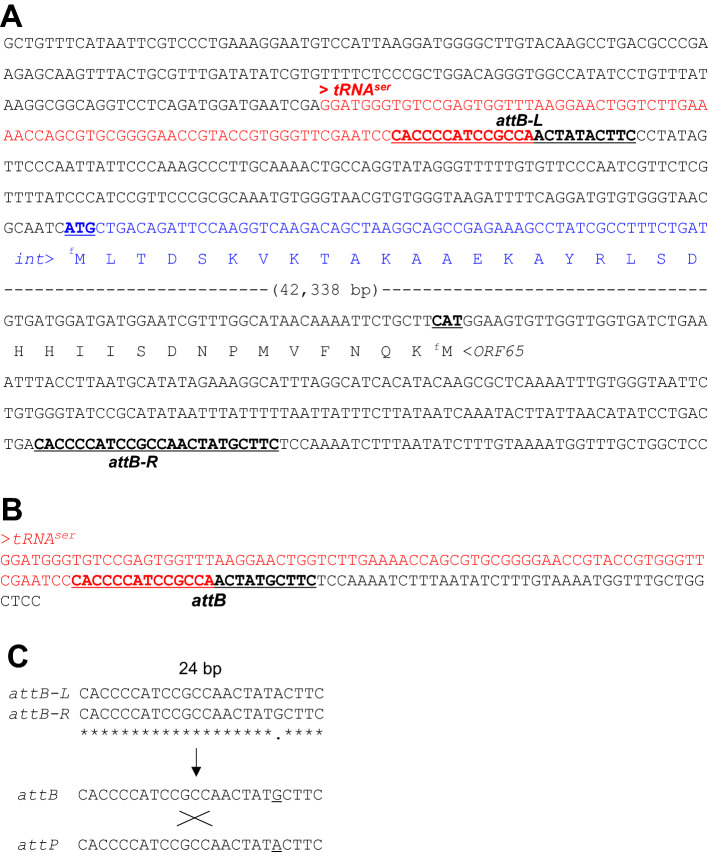
Nucleotide sequences of *att* sites in *A. pasteurianus* NBRC 109446 and phiAP1. (**A**) Bacterial host *att* sites (*attB-L* and *attB-R*) underlined are located in the genomic boundary region between the host and phiAP1. The *tRNA*^*ser*^ gene is shown in red letters. (**B**) The *attB* site in the phiAP1-cured C-27 strain is underlined. The *tRNA*^*ser*^ gene is shown in red letters. (**C**) Comparison of the nucleotide sequences of the *att* sites with a length of 24 bp.

Nucleotide sequences of *attB* sites (*attB-L* and *attB-R*) are generally similar to each other, and they are located in the neighborhood region of phage integrase gene and/or within host *tRNA* gene^21^. We then searched *attB* sites for phiAP1, and found putative *attB-L* (5′-CACCCCATCCGCCAACTATACTTC-3′) and *attB-R* (5′-CACCCCATCCGCCAACTATGCTTC-3′) in the upstream region of the integrase gene (Fig. 2B) and around the 3′ region of *tRNA*^*ser*^ gene (anticodon:TGA), respectively (Fig. 3A). Two *attBs* were 24 bp in total length with a 1 bp difference shown by underline. We also analyzed the *attB* site of the phiAP1-cured region in the C-27 strain, and assigned the following nucleotide sequences 5′-CACCCCATCCGCCAACTATGCTTC-3′ (Fig. 3B) as an *attB*.

We then searched the phage attachment site, *attP*, in the phiAP1 genome. The predicted *attP* sequence had a 1 bp difference to *attB-L* (Fig. 3C). Based on the high nucleotide sequence similarity among attachment sites, it seems likely that Integrase encoded in phiAP1 catalyzes site-specific recombination between *attB* and *attP* to insert the phage genome into the *tRNA*^*ser*^ gene in the host chromosome, and resulting in the generation of a prophage retaining *attB-L* and *attB-R* (Fig. 3C). The integration does not result in disruption of the *tRNA*^*ser*^ gene (Fig. 3A,B).

### Conservation of the phiAP1-like *attB* site in *Acetobacter* spp

**Figure 4:**
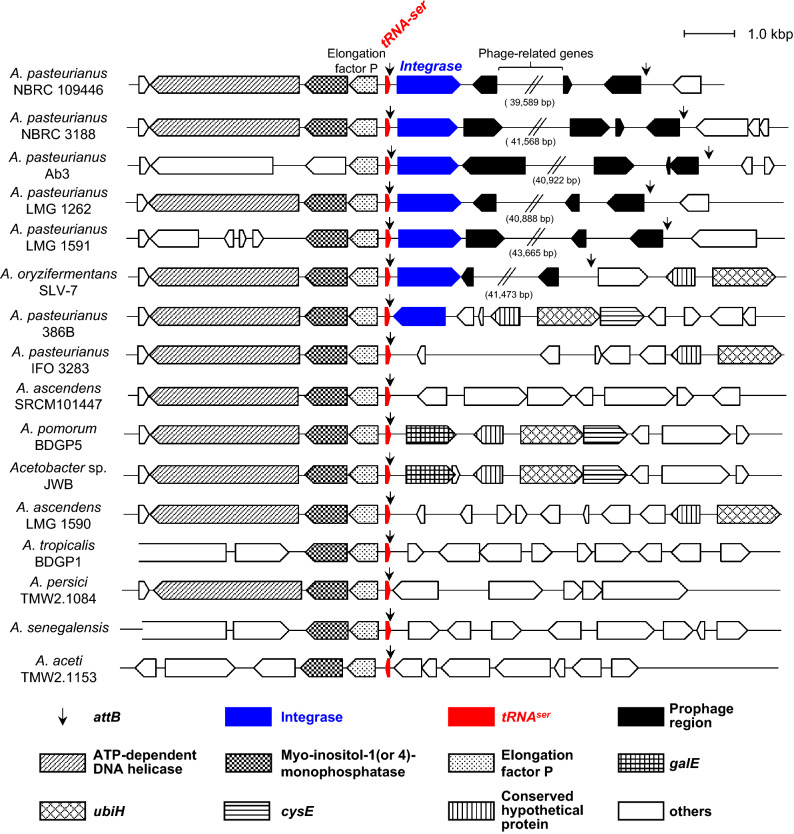
Gene organization of *tRNA*^*ser*^ gene locus in *Acetobacter* spp. The gene compositions and arrangements located in the *tRNA*^*ser*^ locus of *Acetobacter* spp. are shown. The upstream region of *tRNA*^*ser*^ was highly conserved among the genus of *Acetobacter*. Arrowheads indicate the putative *attB* sites. Genes for *tRNA*^*ser*^ and integrase are indicated by red and blue-colored arrowheads, respectively.

To investigate the conservation of phiAP1-like elements, we analyzed the synteny of the *tRNA*^*ser*^ locus in the genome-sequenced *Acetobacter* stains (Fig. 4). The *tRNA*^*ser*^ and an elongation factor P genes are completely conserved in this genus. We found that phiAP1-like elements are located in the region adjacent to *tRNA*^*ser*^ in the four *A. pasteurianus* strains including NBRC 3188, Ab3, LMG 1262*,* LMG 1591, and *A. oryzifermentans* SLV-7. These phiAP1-like elements were predicted by PHAST analysis to form active phage. This result indicates that phiAP1-like elements are distributed and located in the same locus or genomic region across the *A. pasteurianus* strains.

**Figure 5:**
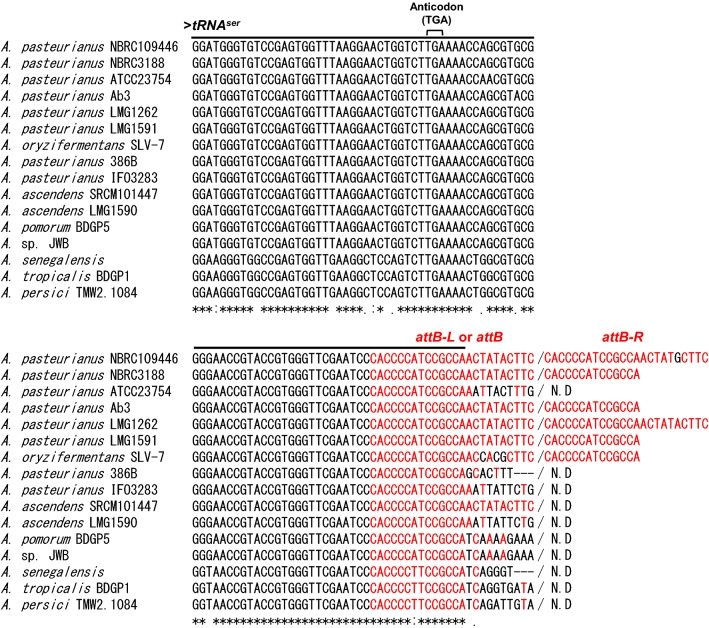
Multiple alignments of *attB* sites in *Acetobacter* spp. Nucleotide sequences of the *tRNA*^*ser*^ gene and its 3’ flanking region were compared with CLUSTAL W (see Materials and Methods). Anticodon (TGA) was located in the position 35 to 37 bp. The coding sequences of the *tRNA*^*ser*^ gene are shown by the line on top of the alignment. Putative *attB-L* and *attB-R* are shown in red text. N.D., not detected. Asterisks, colons, and periods are indicative of completely, highly, and semi-conserved nucleotide sequences, respectively.

In order to predict *attB* sites located in the above conserved region, we compared the nucleotide sequences of the *tRNA*^*ser*^ gene and its 3’-flanking region (Fig. 5). The full length *attB-L* and *attB-R* sequences of *A. pasteurianus* NBRC 109446 were only conserved in *A. pasteurianus* LMG 1262 strain with a 1 bp-substitution. Whereas the *attB* sites in the other four lysogenic strains (*A. pasteurianus* NBRC 3188, Ab3, LMG 1591, and *A. ascendens* SRCM 101447) were predicted as 5’-CACCCCATCCGCCA-3’ with a length of 14 bp. The short *attB-L* site with 14 bp length is also highly conserved in non-lysogenic strains (Fig. 5). This suggests that the short *attB* sites are also recognized by Integrase.

### Infection of *A. pasteurianus* C-27 strain by phiAP1

**Figure 6:**
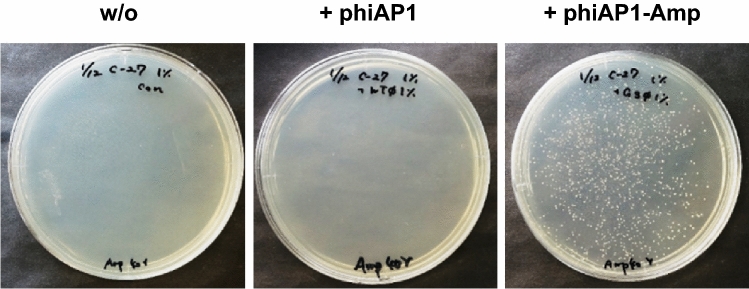
Infective ability of phiAP1 to the *A. pasteurianus* C-27 strain. The phiAP1 and phiAP1-Amp-containing fractions were prepared from *A. pasteurianus* NBRC 109446 wild-type and GMS3 strains cultured with YPG-1% glucose containing MMC. As a recipient cell, the C-27 strain grown in YPG-1% glucose was used. The phage-infected C-27 strains were selected on YPG-1% glucose containing ampicillin at 30 °C for 3 days.

Our genome analysis implies that phiAP1 is able to infect the host cell and integrate its genome into the chromosome via the function of integrase. We then examined the infective ability of phiAP1 with its phiAP1-cured strain C-27. Unexpectedly, we were unable to observe any plaques on confluent lawns of the C-27 strain, despite testing the assay using various phage-fractions prepared from different culture conditions. This result suggests that phiAP1 rapidly and stably integrates into the host genome after infection, and it do not form plaques on host cell lawns by maintaining a stable lysogenic cycle. In order to enable selection of the phiAP1-infected cells, we constructed a modified phiAP1 that carries an ampicillin-resistance gene on its genome (designated as phiAP1-Amp). The phiAP1-Amp particles induced from a host GMS3 strain harboring phiAP1-Amp prophage were observed with TEM analyses (Fig. S2). We then spotted phiAP1-Amp-containing fractions onto the lawns of the C-27 strain, and screened for ampicillin-resistant C-27 strains after 2 days of co-incubation. The C-27 strain grown in the phage-spotted region was harvested with a spatula, and the cell suspended in sterile distilled water was inoculated onto YPG solid medium containing ampicillin. As shown in Fig. 6, the ampicillin-resistant C-27 strains were observed on YPG solid medium supplemented with ampicillin. The specific integration of phiAP1-Amp into the *attB* site was confirmed by PCR analysis. In contrast, no colonies were observed when phiAP1-Amp was co-incubated with the wild-type strain. This result indicates that phiAP1 has the ability to specifically infect the cured host cell, and integrate into the genome.

### Construction of a chromosome-integrative vector, p2096int, for *A. pasteurianus* NBRC 109446

Lysogeny is established by site-specific recombination between the temperate phage genome and bacterial chromosome, and this process is catalyzed by phage integrase. To evaluate the function of phiAP1 integrase and develop a useful molecular tool for AAB research, we constructed a vector capable of integrating into the chromosome (designated as p2096int). The general feature of a chromosome-integrative vector is specific and efficient integration into *attB*, and stable maintenance in the chromosome^21^ which enables the introduction of large gene clusters such as the secondary metabolite biosynthesis gene cluster. This type of vector is also frequently used for genetic complementation experiments, because it’s copy number is identical with the chromosome.

**Figure 7:**
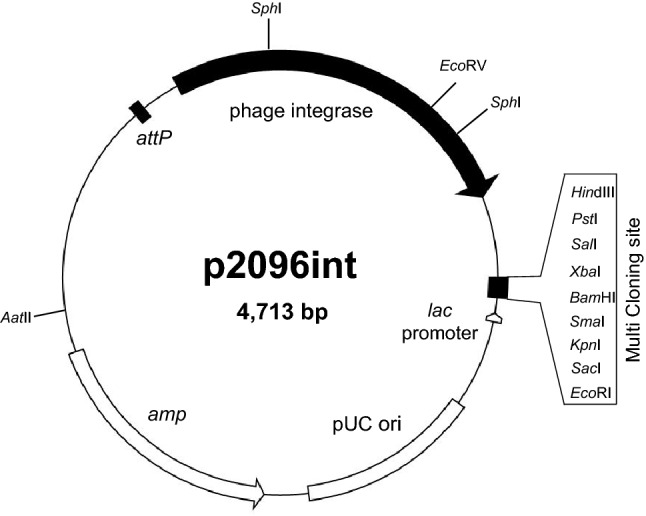
Plasmid map of a chromosome-integrative vector p2096int. p2096int vector contains *attP*, the integrase gene, multiple cloning sites (MCS), *lac* promoter, and an ampicillin-resistance gene. The vector is a pUC-based multicopy number plasmid propagated in *E. coli*, while it is a chromosome-integrative vector in the *A. pasteurianus* C-27 strain.

As shown in Fig. 7, we designed the components of an *E. coli*-*Acetobacter* shuttle vector p2096int carrying the integrase gene, *attP*, pMB1ori (pUC19-based non-replicative in *Acetobacter*), ampicillin-resistance gene, and multiple cloning site (MCS), and *lacZalpha* for blue-white selection. The introduction of p2096int into the phiAP1-cured C-27 strain led to formation of a large number of ampicillin-resistance colonies at 6.1 × 10^3^ cfu (transformants with 1 $\upmu$g DNA) (Table 2). The occurrence of the site-specific recombination between the *attP* and *attB* site was confirmed by PCR analysis. On the other hand, no colony was obtained when p2096int was introduced into the *A. pasteurianus* NBRC 109446 wild-type strain, which suggests the existence of a mechanism preventing multiple phage infection by repressing integrase gene expression from p2096int. These results indicate that phiAP1 integrase is functionally active in *A. pasteurianus*, and p2096int is used as a site-specific chromosome-integrative vector.

**Table 2:** Transformation efficiency with p2096int vector.

Host strains	Transformants/$\upmu$g DNA
*A. pasteurianus* NBRC 109446	0
*A. pasteurianus* C-27 strain	6.1 × 10^3^
*A. pasteurianus* NBRC 3188	2.4 × 10^2^
*A. pasteurianus* ATCC 23754	1.1 × 10^3^

To examine the host range of p2096int, we introduced this vector into total 38 strains of *Acetobacter* spp, 10 strains of *Komagataeibacter* spp, three strains of *Gluconacetobacter* spp, and two strains of *Gluconobacter* spp (Table S2). Two strains of *A. pasteurianus* were found to be recipients at a frequency of 2.4 × 10^2^ cfu (transformants with 1 $\upmu$g DNA) in *A. pasteurianus* NBRC 3188, and 1.1 × 10^3^ cfu in *A. pasteurianus subsp. pasteurianus* ATCC 23754 (Table 2).

We determined the p2096int-integrated site in the two p2096int-accepted strains. In *A. pasteurianus* NBRC 3188, p2096int was integrated at the *attB-L* of the phiAP1-like element (Fig. 4), and the nucleotide sequences and length of *attB-L* were completely identical with that of *A. pasteurianus* NBRC 109446 (Fig. 5). In *A. pasteurianus* ATCC 23754, p2096int was integrated at the 3’ region of *tRNA*^*ser*^ (Fig. 5), and the nucleotide sequences was partially identical with that of *A. pasteurianus* NBRC 109446. In this strain, phiAP1-like element was not found in the intergenic region between Elongation factor P and the upstream gene, which suggests that this strain does not retain phiAP1-like element in this genomic region.

We also analyzed the *attP* site of p2096int vector to determine the essential region for integration. The derivatives of p2096int vector were constructed, which retain the truncated *attP* site (Table 3). The CFU of p2096int-M1 vector retaining 7 bp truncated *attP* was apparently low compared with that of the parental p2096int. The CFU of p2096int-M2 and M3 was more low than that of p2096int-M1. This result suggests that efficient transformation with p2096int requires the 24 bp *attP* with a full length.

**Table 3:** Transformation efficiency with p2096int vector retaining truncated *attB* site.

Name of *attB* site	Nucleotide sequence of *attB* site (5′–3′)^a^	Transformants/$\upmu$g DNA
p2096int	ATCCTGACTGACACCCCATCCGCCAACTATACTTCCCTATAGTTCC	4.76 × 10^3^
p2096int M-1	ATCCTGACTGACACCCCATCCGCCAACTTCCCTATAGTTCCAATTA	1.88 × 10^2^
p2096int M-2	ATCCTGACTGACACCCCATCCGCCATTCCCTATAGTTCCCAATTAT	12.8 × 10
p2096int M-3	ATCCTGACTGACACCCCATCCGCCTTCCCTATAGTTCCCAATTATT	5.1 × 10

^a^ Nucleotide sequences of *attB* were shown by underline.

## Discussion

Our study showed that MMC-inducible temperate phages are widely distributed in the bacterial group of AAB used for industrial vinegar fermentation. The phage particles observed by TEM analyses were classified into myoviride-type, and the six of the genome structures were determined. Our study also revealed the presence of an infectious temperate phage, phiAP1, derived from *A. pasteurianus*. The phiAP1-like elements were highly conserved in the genomes of *A. pasteurianus* strains, suggesting that phiAP1 is a major prophage in this species. Our comparative analysis identified the *att* sites required for site-specific integration of the phage genome into the host chromosome catalyzed by phiAP1 integrase. We also applied the integrase gene to development of a chromosome-integrative vector p2096int, which was efficiently and specifically integrated into three *A. pasteurianus* strains. This study provides fundamental information related to and application of temperate phages in AABs.

Our wide investigation of temperate phages derived from a total of 177 AAB strains showed that about 7.4% of AABs possess MMC-inducible temperate phages within their genomes. The requirement of MMC suggests that phage induction is linked to the SOS response of the host responsible for DNA repair. On the other hand, PHAST analysis suggests that 55% of AAB strains retain at least one of temperate phages in their genome. These suggest that SOS response of the host responsible for DNA repair, induced by physiological and chemical mutagens such as UV, MMC, and reactive oxygen species^9^, is a major pathway for phage induction. The large difference in the number between our analysis and genomic information also suggests that phage induction is required other stress generating in the acetic acid fermenter or AAB-dwelling environment. As far as we know, stress treatments other than MMC for inducing phage have not been found in this group of bacteria. Further study on stress for this group of bacteria generating in the fermenter or its actual dwelling environment will be to understanding the interaction of prophage and environments, and which is useful for the stable fermentation.

Morphological analyses using TEM supports that all of the AAB phages detected in this study belong to the myoviride type, which are found in a wide range of bacteria and archaea. Myoviridae type phages are non-enveloped with a head–tail, and their genome is a linear, double-stranded DNA, around 33 to 244 kb in length (https://viralzone.expasy.org/). Myoviridae type phages are generally lytic type, but temperate types have also been identified (https://viralzone.expasy.org/). In our study, the characterized phiAP1 is a temperate phage retaining a circular genome as supported by the possession of integrase.

Our analysis supports that phiAP1 is an active temperate phage, and most widely distributed-type prophage in *A. pasteurianus,* based on the following: (i) phiAP1 has the ability to infect its cured C-27 strain, (ii) phiAP1-like elements are found in many species of *A. pasteurianus* with similar gene composition and arrangement, (iii) the integrases have a high similarity to each other, and (iv) the sequences of *attB-L* and *attB-R* are conserved. In the five phiAP1-like elements (Fig. 2B), the proteins encoded in the central region are phage-related proteins, and many of them are found in other phage genomes, whereas many short ORFs flanked by the integrase gene are short compared to their bacterial homologs. There is a possibility that the short ORFs are remaining junk DNA generated during infection. Generally, it is known that phage genomes retain a mosaic structure consisting of the different origin phage genome, generating a high diversity of phages in nature^23^. The high conservation of gene composition and arrangement in the phiAP1-like elements supports that phiAP1s have an identical origin during evolutionary history.

Our study revealed that the site-specific integration of the phiAP1 genome into the host *attB* site was reproducible when phiAP1 was co-cultured with the C-27 strain. Meanwhile, plaque formation by phiAP1 was never observed under any culture condition used in this study. This indicates that phiAP1 is immediately and stably integrated into the host genome after infection. In λ phage in *E. coli*, CI repressor protein belonging to the Xre family inhibits the lytic pathway maintaining the prophage state^24^. Pfam search predicted that ORF11, 12, 17, 18 of phiAP1 belongs to the Xre family, which suggests a possibility that these ORFs may control the switching between lytic and lysogenic states. The expression level of these ORFs could be involved in the inability of plaque formation by phiAP1.

The *attB* site of *A. pasteurianus* NBRC 109446 was suggested as 5′-CACCCCATCCGCCAACTATACTTC-3′ with a 24 bp length. A similar sequence with identical length was also conserved in the phiAP1-like element of *A. pasteurianus* LMG 1262 (Fig. 5). In contrast, other *A. pasteurianus* strains retain short sequences of the *attB* site. The difference in *attB* recognition might be caused by integrase amino acid sequence differences; however, the integrases encoded in the phiAP1-like elements have a high similarity to each other, except for the end of the C-terminus. Namely, the integrases of *A. pasteurianus* NBRC 109446 and LMG 1262 retain an identical extended 21 amino acid sequence in the C-terminus, while other integrases have no extended region (Fig. S3). This suggests that the extended sequence is involved in the recognition of the long *attB* sequence. Further study is required for understanding the recognition mechanism of phiAP1 integrases.

Transformation efficiency of a chromosome-integrative vector p2096int in the three *Acetobacter* strains is sufficient for the general cloning experiment (Table 2). The site-specific recombination of p2096int into the *attB* site was also reproducibly confirmed. However, an expansion of host range is required to improve the versatility in AAB group bacteria. In $\lambda$ phage, it is known that CI repressor serves as an immune mechanism for preventing multiple phage infections. This suggests that the CI-like repressors, ORF11, 12, 17, 18, encoded on the phiAP1 genome, negatively acts on the expression or function of phiAP1 integrase to prevent the integration of p2096int into the chromosome; this may be a reason why the parental strain of *A. pasteurianus* NBRC 109446 was unacceptable for p2096int. However, *A. pasteurianus* NBRC 3188, an acceptable strain for p2096int, retains three CI-like repressors, ORF10, 16, 17, in the phiAP-like elements. In another p2096int-acceptable *A. pasteurianus* ATCC 23754, a phiAP-like element is not found in the *tRNA* locus, suggesting that this strain is non-lysogenic. Further molecular genetic analysis on the three acceptable strains, and improvement of p2096int such as replacement of integrase promoter with a constitutive one will help to understand the host-specificity of the p2096int vector, and contribute to improving the versatility of the vector.

In this study, we also obtained the prophage cured strain, C-27 in *A. pasteriuanus* NBRC 109446. We believe that the C-27 strain is a useful host based on the following reasons: (i) The strain is “prophage-free safety host” because phage-induced cell lysis does not occur when DNA is damaged. (ii) The cured strain has an equal ability in acetic acid fermentation with the wild-type strain (unpublished data). (iii) If the p2096int vector is modified to a cosmid, it can carry large gene clusters for secondary metabolite biosynthesis. Therefore, the C-27 strain could be a host for the stable production of useful metabolites. This strain, together with the p2096int vector, can be widely used in basic and applied research.

## Materials and methods

### Bacteria, plasmids, oligonucleotides, and culture media

Acetic acid bacteria (AAB) used in this study are listed in Table S2, and were obtained from culture collections including JCM, NBRC, IAM, NRIC, ATCC, and DSM. *Escherichia coli* HST08 for general cloning host, pUC19 as a cloning vector, and pMD19 as a TA cloning vector were purchased from Takara Bio Inc. (Shiga, Japan). An ampicillin-resistant pMV24 plasmid was used as an *Acetobacter* spp.-*E. coli* shuttle vector^25^. Oligonucleotide primers used for PCR are summarized in Table S5. AABs were grown at 30 °C in YPG medium [containing (per liter): yeast extract, 5 g; hipolypeptone, 3 g; glucose, 30 g]. *E. coli* was grown in Luria–Bertani (LB) medium. For preparation of solid medium, 1.5% agar was added. To select transformants of *E. coli* and *Acetobacter* spp., ampicillin was added at 40 $\upmu$g/mL. All chemicals and enzymes used were obtained from Wako Pure Chemical (Osaka, Japan) and Takara Bio Inc., respectively, unless otherwise indicated.

### Selection of lysogenic AAB stains

To select lysogens from a total of 177 AAB strains (Table S2) obtained from culture collections, AABs were grown on YPG solid medium containing 0.2 to 4.0 $\upmu$g/mL mitomycin C (MMC) at 28 °C for 4 days. AAB strains exhibiting MMC sensitivity on solid medium were subjected to secondary selection with YPG liquid medium containing 0.2 to 4.0 $\upmu$g/mL MMC with shaking culture at 110 rpm. AAB strains in which growth was inhibited by MMC were regarded as candidates for lysogens.

### Preparation of fractions containing temperate phages

To observe temperate phages induced from the MMC-treated AABs, phage-containing fractions were prepared by concentrating the supernatant of AAB culture broth. All of the tested AABs were cultured in YPG liquid medium at 30 °C overnight with shaking at 160 rpm. 1 mL of the pre-cultured broth was inoculated to 100 mL of YPG liquid medium supplemented with 4.0 $\upmu$g/mL MMC in a 500 ml Erlenmeyer flask with baffle. Culture was performed at 30 °C for 48 h in a rotary shaker at 160 rpm. After removal of the grown cells by centrifugation and filtration with 0.22 $\upmu$m pore size filter, 25 mL of 30% polyethylene glycol (PEG) 8,000 solution containing 5 M NaCl was added to the supernatant, and then incubated at 4 °C overnight. The samples were transferred into 40PA centrifuge tubes (Hitachi), and centrifuged at 30,000 rpm at 4 °C for 2 h with himac CP100 MX ultracentrifuge. After removing the supernatant, the precipitant was suspended with 100–200 $\upmu$L of 0.85% saline solution or distilled water, resulting in a 500 to 1000-fold concentration compared to the initial culture broth.

### Visualization of phages with TEM analyses

Phage-containing fraction (3 $\upmu$L) was mixed with the same volume of two-fold diluted EM stainer with distilled water (Nisshin-EM. Co., Ltd., Tokyo, Japan), and incubated for 1 min at room temperature for negative staining. The mixture was spotted on collodion membrane 200-mesh (Nisshin-EM), and incubated for 90 secs at room temperature. Excess staining solution was removed with whatman filter paper. Specimens were examined with a JEM-1010 (JEOL Ltd., Tokyo, JAPAN) operating at 100 kV according to manufacturer’s instruction, and micrographs were developed on ELESCOPE FG film 8.2 × 11.8 cm (Fujifilm, Tokyo, Japan).

### Determination of whole genome sequences of lysogenic phages and bioinformatics analyses

The phage fractions derived from *A. pasteurianus* NBRC 109446, *A. orleanensis* NBRC 3170, *Acetobacter* sp. ATCC 21760, *K. xylinus* NBRC 13772, *K. xylinus* NBRC 13773, and *K. maltaceti* NBRC 14815 were prepared as described above. The fractions were treated with 20 $\upmu$g/mL DNase I (Sigma-Aldrich Corp, St. Louis, MO, USA) and 10 $\upmu$g/mL RNase (Sigma-Aldrich) at 37 °C for 30 min. Their phage genomic DNAs were purified with Phage DNA isolation kit (Norgen Biotek Corp., Ontario, Canada) according to manufacturer’s instruction. Quality and quantity of the purified phage genomic DNAs were verified with GeneQuant 100 (GE Healthcare UK Ltd, Buckinghamshire, England), and agarose gel electrophoresis analyses. Genome sequence analysis with next generation sequencing was performed using Illumina MiSeq with 150-bp paired-end reads by Hokkaido System Science Co. Ltd. (Sapporo, Japan). DFAST^26^, RAST^27^, 2ndFind (http://biosyn.nih.go.jp/2ndfind/), PHAST^15^ was used for the annotation of phage genomes. Genetyx ver. 13 (GENETYX Corporation, Tokyo, JAPAN) was used for local BLAST searches using draft genome sequences. Schematic representation of gene organization was drawn with drawGeneArrows3 (http://www.ige.tohoku.ac.jp/joho/index.html). The prediction of the *tRNA* gene was performed with tRNAscan-SE^28^. Multiple alignment was carried out by CLUSTAL W^29^.

### Accession numbers

The nucleotide sequences determined in this study have been registered to GenBank under the accession numbers LC644972 for phiAP1 of *A. pasteurianus* NBRC 109446, LC644971 for phiAO1 of *A. orleanensis* NBRC 3170, LC644973 for phiAX1 of *Acetobacter* sp. ATCC 21760, LC644975 for phiKX1 of *K. xylinus* NBRC 13772, LC644976 for phiKX2 of *K. xylinus* NBRC 13773, and LC644974 for phiKM1 of *K. maltaceti* NBRC 14815.

### Construction of a phiAP1-cured C-27 strain in *A. pasteurianus* NBRC 109446

The wild-type strain of *A. pasteurianus* NBRC 109446 was grown in YPG liquid medium containing 0.05% to 0.1%Tween 80 at 28 °C with shaking at 160 rpm for 16 to 24 h. The propagated cells were diluted with sterile distilled water, and spread on YPG solid medium containing MMC to form single colonies. To identify the prophage-cured stains, the prophage region of phiAP1 was detected by colony PCR with primer set M30-ck3F/M30-ck3R, which anneals to the inner region of the phiAP1 genome. To confirm whether the cured strain lost the prophage, boundary regions between host and prophage genomes were analyzed by PCR using primer set M30-ck1F/M30-ck1R and M30-ck2F/M30-ck2R.

To construct p2096int-M1, p2096int-M2, and p2096int-M3, inverse PCR was used. Each DNA fragment containing a full length region was amplified by inverse PCR with primer pair, 2096int-M-R/2096int-M1-L for p2096int-M1, 2096int-M-R/2096int-M2-L for p2096int-M2, and 2096int-M-R/2096int-M3-L for p2096int-M3. The purified amplicons were self-ligated with T4 DNA Ligase. The constructed vectors were sequenced by an ABI3100 sequencer (Thermo Fisher Scientific) or Eurofins Genomics K.K. (Tokyo, Japan). The constructed p2096int derivatives were introduced into *A. pasteurianus* C-27 strain by electroporation as described above.

### Construction of ampicillin-resistant lysogenic host of *A. pasteurianus* NBRC 109446

To construct *A. pasteurianus* NBRC 109446 retaining an ampicillin-resistant gene in the prophage region, an ampicillin(Amp)-resistant pGMS3 vector harboring the 1,365 bp region homologous with the internal sequences of a gene encoding a putative serine peptidase (ORF37 in Fig. 3 and Table S4) was constructed as follows. The DNA fragment containing the internal region of ORF37 gene within phiAP1 was generated by PCR with primer set Dis19188F/Dis20547R. The amplicon was then directly cloned into an Amp-resistant TA vector pMD19, yielding the pGMS3 vector. *A. pasteurianus* NBRC 109446 wild-type was transformed with pGMS3 by electroporation, and the recovery culture allowing the expression of Amp-resistant gene was performed at 30 °C for 6 h. The single-crossover strains exhibiting Amp resistance were selected on YPG solid medium containing 40 $\upmu$g/mL ampicillin. The proper single-crossover recombination to the *attB* site was confirmed by PCR with an appropriate primer set. The constructed strain was designated as the *A. pasteurianus* GMS3 strain, and its Amp-resistant gene-retaining phage was designated as phiAP1-Amp.

### Infection of the phiAP1-cured C-27 strain with phiAP1

To prepare the phiAP1-Amp-containing fraction, *A. pasteurianus* GMS3 strain was cultured in the presence of MMC, and the cell-free supernatant of culture broth was concentrated with 30% PEG 8,000 solution containing 5 M NaCl as described above. The phiAP1-Amp-containing fraction was spotted onto the lawn of the C-27 strain grown on YPG solid medium, and the plates were incubated at 30 °C for 48 h. The C-27 strain grown in the phage-spotted region was harvested with a spatula, and the cell suspended in sterile distilled water was inoculated onto YPG solid medium containing 40 $\upmu$g/mL ampicillin.

### Construction of a chromosome-integrative vector p2096int for *A. pasteurianus* C-27 strain

A 1,210 bp DNA fragment containing *attP*, a promoter region of *integrase*, as well as the N-terminal region of *integrase*, was amplified by PCR with primer set 2096int-F1/2096int-R1. The 863 bp DNA fragment containing the C-terminal region of the integrase gene was amplified by PCR with 2096int-F2/2096int-R2 using a synthetic DNA of C-terminal region of the integrase gene as template. An 840 bp DNA fragment containing the *attP* and integrase gene of phiAP1 was synthesized by Eurofins Genomics K.K. (Tokyo, Japan) to remove the restriction enzyme sites used in the multiple cloning site. The above two DNA fragments were fused by overlapping PCR using primer set 2096int-F1/2096int-R2. The amplicon was cleaved with *Nde*I, and inserted into the same site of pUC19, yielding a p2096int that was 4,723 bp in total length. The constructed p2096int was introduced into AAB strains by electroporation. Transformants were selected on YPG solid medium containing 40 $\upmu$g/mL Amp, and proper integration was confirmed by PCR with an appropriate primer set.

### Determination of *attB* sites in *A. pasteurianus* NBRC 3188 and *A. pasteurianus subsp. pasteurianus* ATCC 23754

The *attB* site of *A. pasteurianus* NBRC 3188 was predicted by homology search with the draft genomic information (GenBank accession number: NZ_BDES00000000) using local BLAST in Genetyx. To predict the nucleotide sequence of the *attB* site in *A. pasteurianus* ATCC 23754, the intergenic regions of Elongation factor P and the upstream ORF66 were amplified by PCR with primer pair, tRNASer_attB_F2/tRNASer_attB_R4, which were constructed based on nucleotide sequences conserved among *Acetobacter* spp. The amplicons were cloned into pMD19 vector by TA cloning, and nucleotide sequences of the resultant clones were determined by sequencing service of Eurofins Genomics or using an ABI 3100 Genetic analyzer (Thermo Fisher Scientific, Waltham, MA, USA).

## Supplementary Information

Supplementary Information 1.

Supplementary Information 2.

Supplementary Information 3.

Supplementary Information 4.

Supplementary Information 5.

Supplementary Information 6.

Supplementary Information 7.

Supplementary Information 8.
