# Supplementary material for: Distribution and genome structures of temperate phages in acetic acid bacteria
Source: Sci Rep. 2021 Nov 3;11:21567. doi: 10.1038/s41598-021-00998-w (PMC8566455; doi:10.1038/s41598-021-00998-w)
Supplement: Supplementary file 1 — Supplementary Information 1. [file 41598_2021_998_MOESM1_ESM.pptx]

## Slide 1
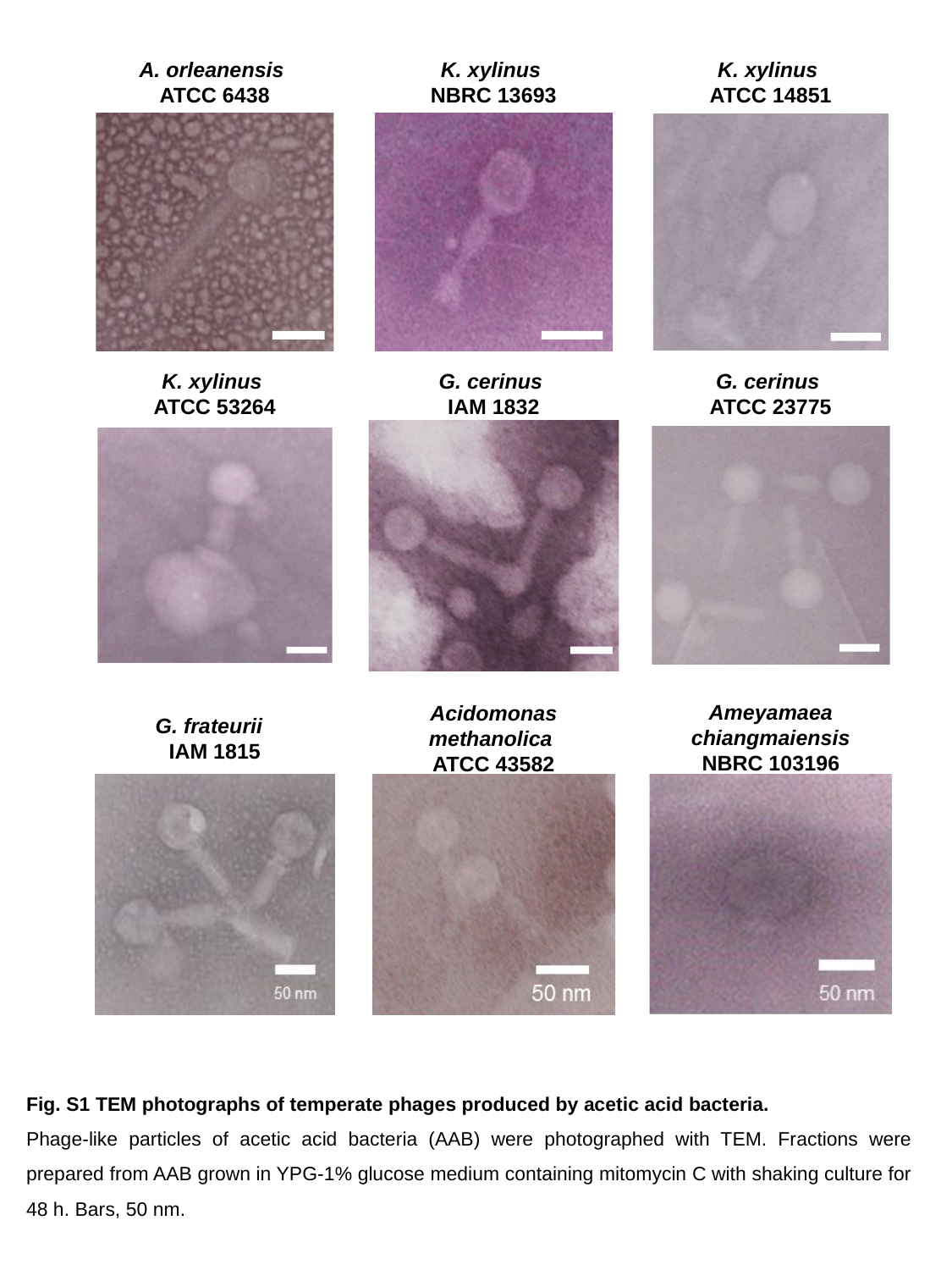

A. orleanensis
ATCC 6438
K. xylinus
NBRC 13693
K. xylinus
ATCC 14851
G. cerinus
IAM 1832
G. cerinus
ATCC 23775
K. xylinus
ATCC 53264
Ameyamaea chiangmaiensis NBRC 103196
Acidomonas methanolica
ATCC 43582
G. frateurii
IAM 1815
Fig. S1 TEM photographs of temperate phages produced by acetic acid bacteria.
Phage-like particles of acetic acid bacteria (AAB) were photographed with TEM. Fractions were prepared from AAB grown in YPG-1% glucose medium containing mitomycin C with shaking culture for 48 h. Bars, 50 nm.
