# Supplementary material for: Distribution and genome structures of temperate phages in acetic acid bacteria
Source: Sci Rep. 2021 Nov 3;11:21567. doi: 10.1038/s41598-021-00998-w (PMC8566455; doi:10.1038/s41598-021-00998-w)
Supplement: Supplementary file 2 — Supplementary Information 2. [file 41598_2021_998_MOESM2_ESM.pptx]

## Slide 1
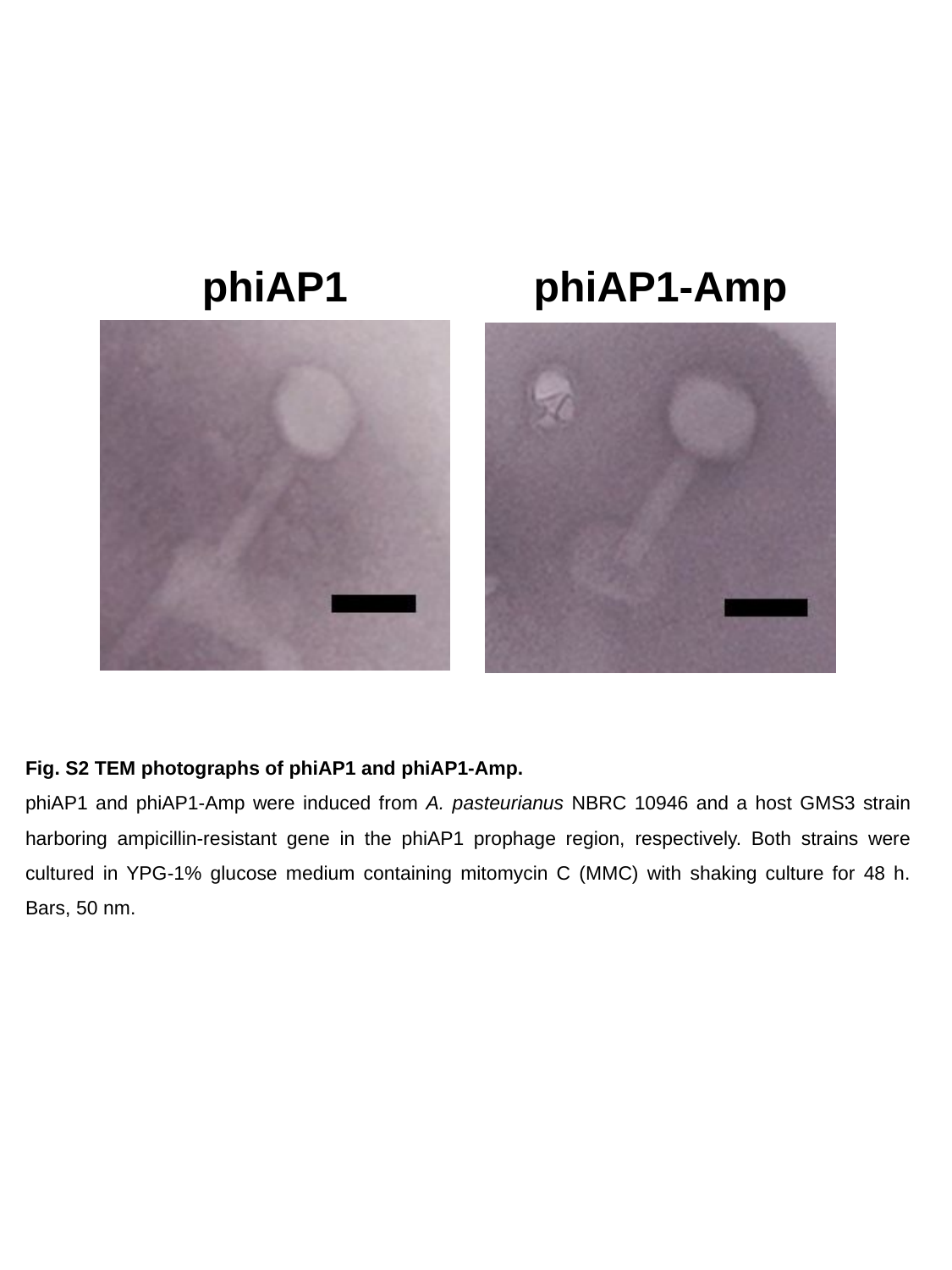

phiAP1-Amp
phiAP1
Fig. S2 TEM photographs of phiAP1 and phiAP1-Amp.
phiAP1 and phiAP1-Amp were induced from A. pasteurianus NBRC 10946 and a host GMS3 strain harboring ampicillin-resistant gene in the phiAP1 prophage region, respectively. Both strains were cultured in YPG-1% glucose medium containing mitomycin C (MMC) with shaking culture for 48 h. Bars, 50 nm.
