# Supplementary material for: Distribution and genome structures of temperate phages in acetic acid bacteria
Source: Sci Rep. 2021 Nov 3;11:21567. doi: 10.1038/s41598-021-00998-w (PMC8566455; doi:10.1038/s41598-021-00998-w)
Supplement: Supplementary file 3 — Supplementary Information 3. [file 41598_2021_998_MOESM3_ESM.pptx]

## Slide 1
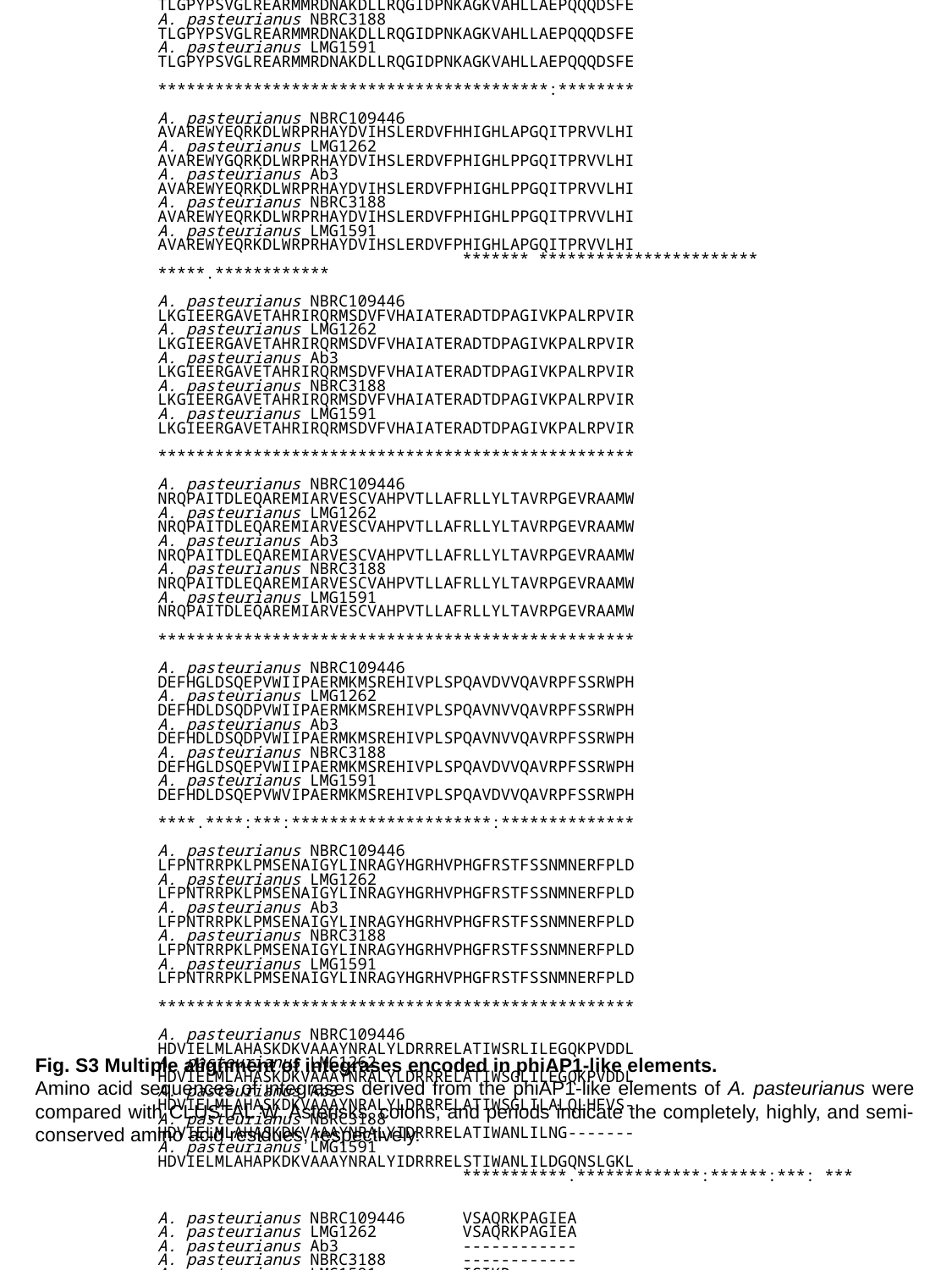

A. pasteurianus NBRC109446 MLTDSKVKTAKAAEKAYRLSDSEGLFVHVMPTGKKFWRLRYRQQGKEQTL
A. pasteurianus LMG1262 MLTDSKVKTAKAAEKAYRLSDSEGLFVHVMPTGKKFWRLRYRQQGKEQTL
A. pasteurianus Ab3 MLTDSKVKTAKAAEKAYRLSDSEGLFVHVMPTGKKFWRLRYRQHGKEQTL
A. pasteurianus NBRC3188 MLTDSKVKTAKAAEKAYRLSDSEGLFVHVMPTGKKFWRLRYRQQGKEQTL
A. pasteurianus LMG1591 MLTDSKVKTAKAAEKAYRLSDSEGLFVHVMPTGKKFWRLRYRQQGKEQTL
 *******************************************:******
A. pasteurianus NBRC109446 TLGPYPSVGLREARMMRDNAKDLLRQGIDPNKAGKVAHLLAEPQQQDSFE
A. pasteurianus LMG1262 TLGPYPSVGLREARMMRDNAKDLLRQGIDPNKAGKVAHLLADPQQQDSFE
A. pasteurianus Ab3 TLGPYPSVGLREARMMRDNAKDLLRQGIDPNKAGKVAHLLAEPQQQDSFE
A. pasteurianus NBRC3188 TLGPYPSVGLREARMMRDNAKDLLRQGIDPNKAGKVAHLLAEPQQQDSFE
A. pasteurianus LMG1591 TLGPYPSVGLREARMMRDNAKDLLRQGIDPNKAGKVAHLLAEPQQQDSFE
 *****************************************:********
A. pasteurianus NBRC109446 AVAREWYEQRKDLWRPRHAYDVIHSLERDVFHHIGHLAPGQITPRVVLHI
A. pasteurianus LMG1262 AVAREWYGQRKDLWRPRHAYDVIHSLERDVFPHIGHLPPGQITPRVVLHI
A. pasteurianus Ab3 AVAREWYEQRKDLWRPRHAYDVIHSLERDVFPHIGHLPPGQITPRVVLHI
A. pasteurianus NBRC3188 AVAREWYEQRKDLWRPRHAYDVIHSLERDVFPHIGHLPPGQITPRVVLHI
A. pasteurianus LMG1591 AVAREWYEQRKDLWRPRHAYDVIHSLERDVFPHIGHLAPGQITPRVVLHI
 ******* *********************** *****.************
A. pasteurianus NBRC109446 LKGIEERGAVETAHRIRQRMSDVFVHAIATERADTDPAGIVKPALRPVIR
A. pasteurianus LMG1262 LKGIEERGAVETAHRIRQRMSDVFVHAIATERADTDPAGIVKPALRPVIR
A. pasteurianus Ab3 LKGIEERGAVETAHRIRQRMSDVFVHAIATERADTDPAGIVKPALRPVIR
A. pasteurianus NBRC3188 LKGIEERGAVETAHRIRQRMSDVFVHAIATERADTDPAGIVKPALRPVIR
A. pasteurianus LMG1591 LKGIEERGAVETAHRIRQRMSDVFVHAIATERADTDPAGIVKPALRPVIR
 **************************************************
A. pasteurianus NBRC109446 NRQPAITDLEQAREMIARVESCVAHPVTLLAFRLLYLTAVRPGEVRAAMW
A. pasteurianus LMG1262 NRQPAITDLEQAREMIARVESCVAHPVTLLAFRLLYLTAVRPGEVRAAMW
A. pasteurianus Ab3 NRQPAITDLEQAREMIARVESCVAHPVTLLAFRLLYLTAVRPGEVRAAMW
A. pasteurianus NBRC3188 NRQPAITDLEQAREMIARVESCVAHPVTLLAFRLLYLTAVRPGEVRAAMW
A. pasteurianus LMG1591 NRQPAITDLEQAREMIARVESCVAHPVTLLAFRLLYLTAVRPGEVRAAMW
 **************************************************
A. pasteurianus NBRC109446 DEFHGLDSQEPVWIIPAERMKMSREHIVPLSPQAVDVVQAVRPFSSRWPH
A. pasteurianus LMG1262 DEFHDLDSQDPVWIIPAERMKMSREHIVPLSPQAVNVVQAVRPFSSRWPH
A. pasteurianus Ab3 DEFHDLDSQDPVWIIPAERMKMSREHIVPLSPQAVNVVQAVRPFSSRWPH
A. pasteurianus NBRC3188 DEFHGLDSQEPVWIIPAERMKMSREHIVPLSPQAVDVVQAVRPFSSRWPH
A. pasteurianus LMG1591 DEFHDLDSQEPVWVIPAERMKMSREHIVPLSPQAVDVVQAVRPFSSRWPH
 ****.****:***:*********************:**************
A. pasteurianus NBRC109446 LFPNTRRPKLPMSENAIGYLINRAGYHGRHVPHGFRSTFSSNMNERFPLD
A. pasteurianus LMG1262 LFPNTRRPKLPMSENAIGYLINRAGYHGRHVPHGFRSTFSSNMNERFPLD
A. pasteurianus Ab3 LFPNTRRPKLPMSENAIGYLINRAGYHGRHVPHGFRSTFSSNMNERFPLD
A. pasteurianus NBRC3188 LFPNTRRPKLPMSENAIGYLINRAGYHGRHVPHGFRSTFSSNMNERFPLD
A. pasteurianus LMG1591 LFPNTRRPKLPMSENAIGYLINRAGYHGRHVPHGFRSTFSSNMNERFPLD
 **************************************************
A. pasteurianus NBRC109446 HDVIELMLAHASKDKVAAAYNRALYLDRRRELATIWSRLILEGQKPVDDL
A. pasteurianus LMG1262 HDVIELMLAHASKDKVAAAYNRALYLDRRRELATIWSGLILEGQKPVDDL
A. pasteurianus Ab3 HDVIELMLAHASKDKVAAAYNRALYLDRRRELATIWSGLILALQLHFVS-
A. pasteurianus NBRC3188 HDVIELMLAHASKDKVAAAYNRALYIDRRRELATIWANLILNG-------
A. pasteurianus LMG1591 HDVIELMLAHAPKDKVAAAYNRALYIDRRRELSTIWANLILDGQNSLGKL
 ***********.*************:******:***: ***
A. pasteurianus NBRC109446 VSAQRKPAGIEA
A. pasteurianus LMG1262 VSAQRKPAGIEA
A. pasteurianus Ab3 ------------
A. pasteurianus NBRC3188 ------------
A. pasteurianus LMG1591 ISIKR-------
 .* *
Fig. S3 Multiple alignment of integrases encoded in phiAP1-like elements.
Amino acid sequences of integrases derived from the phiAP1-like elements of A. pasteurianus were compared with CLUSTAL W. Asterisks, colons, and periods indicate the completely, highly, and semi-conserved amino acid residues, respectively.
