# Supplementary material for: Distribution and genome structures of temperate phages in acetic acid bacteria
Source: Sci Rep. 2021 Nov 3;11:21567. doi: 10.1038/s41598-021-00998-w (PMC8566455; doi:10.1038/s41598-021-00998-w)
Supplement: Supplementary file 4 — Supplementary Information 4. [file 41598_2021_998_MOESM4_ESM.docx]

**Table S1 Distribution of temperate phage-like elements in the genomes of AAB**

| **Bacterial hosts** | | | | **Temperate phage-like elements** | | | |
| --- | --- | --- | --- | --- | --- | --- | --- |
| **Genus** | **Species**  **(GenBank No.)** | **Isolation source^a^** | **(Locality of Source / Country of Origin^a^)** | **Localization** | **Type^b^** | **Size (kbp)** | **Position in Genome**  **(bp)** |
| *Acetobacter* | *aceti* NBRC 14818 | Alcohol turned to vinegar | - / - | Chromosome | Incomplete | 6.3 | 2997584-3003901 |
|  | *aceti* TMW2.1153  (CP014692) | Water kefir | Freising / Germany | Chromosome | Intact | 40.6 | 595181-635839 |
|  |  |  |  |  | Intact | 25.1 | 773148-798327 |
|  |  |  |  |  | Incomplete | 26.8 | 3395852-3422730 |
|  |  |  |  |  | Incomplete | 11.0 | 3590313-3601378 |
|  | *ascendens* SRCM101447  (NZ_CP015164, NZ_CP015167.1) | Food | - / South Korea | Chromosome | incomplete | 17.7 | 135027-152779 |
|  |  |  |  |  | incomplete | 7.9 | 1381189-1389102 |
|  |  |  |  |  | questionable | 29.2 | 1901991-1931193 |
|  |  |  |  | Plasmid | incomplete | 13.9 | 24318-38220 |
|  | *oryzifermentans* SLV-7  (CP011120-CP011123) | Vinegar | Gangwon / South Korea | Chromosome | Incomplete | 33.6 | 597058-630720 |
|  |  |  |  |  | Incomplete | 17.7 | 655401-673177 |
|  |  |  |  |  | Incomplete | 8.2 | 1741260-1749531 |
|  |  |  |  |  | Questionable | 15.6 | 1961405-1977102 |
|  |  |  |  | Plasmid | Incomplete | 7.4 | 20657-28100 (116 Kbp unnamed plasmid) |
|  |  |  |  |  | Incomplete | 15.6 | 7223-22870 (25 Kbp unnamed plasmid) |
|  | *pasteurianus* NBRC 3188  (NZ_BDES00000000.1) | - | - / - | Chromosome | Incomplete | 29.4 | 106943-136375 |
|  |  |  |  |  | Incomplete | 17.7 | 461772-479503 |
|  |  |  |  |  | Incomplete | 18.4 | 1821766-1840220 |
|  |  |  |  |  | Intact | 42.2 | 1848560-1890794 |
|  | *pasteurianus* Ab3  (CP012111.1) | Vinegar factory | Huzhou / China | Chromosome | Incomplete | 12.3 | 1344160-1356465 |
|  |  |  |  |  | Incomplete | 17.6 | 1414224-1431874 |
|  |  |  |  |  | Intact | 34.6 | 2738217-2772822 |
|  | *pasteurianus* LMG 1591  (NZ_CP015168.1) | - | - / Netherlands | Chromosome | incomplete | 18.7 | 172000-190700 |
|  |  |  |  |  | incomplete | 40.5 | 1620406-1660990 |
|  |  |  |  |  | Intact | 42.7 | 1636206-1678981 |
|  |  |  |  |  | questionable | 27.8 | 2018069-2045933 |
|  | *pasteurianus* 386B (NC_021991.1) | Cocoa bean heap fermentation | - / Ghana | Chromosome | incomplete | 14.7 | 380383-395099 |
|  |  |  |  |  | incomplete | 17.7 | 2093559-2111307 |
|  | *pasteurianus* IFO 3283-01  (AP011121-AP011127) | Film in fermentor of rice vinegar | Aichi / Japan | Chromosome | Incomplete | 19.4 | 538999-558409 |
|  |  |  |  |  | Intact | 51.6 | 2164321-215942 |
|  | *pasteurianus* subsp. *ascendens* LMG 1590 (CP015164-CP015167) | - | - / Netherlands | Chromosome | Questionable | 22.3 | 130429-152779 |
|  |  |  |  |  | Incomplete | 6.8 | 1381189-1388077 |
|  |  |  |  |  | Incomplete | 22.0 | 1877785-1899784 |
|  |  |  |  |  | Questionable | 29.2 | 1901991-1931193 |
|  |  |  |  |  | Incomplete | 16.8 | 2003360-2020169 |
|  |  |  |  |  | Incomplete | 6.7 | 2057092-2063823 |
|  |  |  |  |  | Incomplete | 16.7 | 2204954-2221702 |
|  |  |  |  |  | Incomplete | 21.3 | 2369271-2390607 |
|  |  |  |  | Plasmid | Incomplete | 20.8 | 24766-45609 (46 Kbp unnamed plasmid) |
|  |  |  |  |  | Incomplete | 13.9 | 24318-38220 (43 Kbp unnamed plasmid) |
|  | *persici* TMW2.1084 (CP014687-CP014688) | Water kefir | Freising / Germany | Chromosome | Incomplete | 9.0 | 39958-49023 |
|  |  |  |  |  | Incomplete | 32.0 | 1722199-1754249 |
|  |  |  |  |  | Incomplete | 16.5 | 2480050-2496558 |
|  | *pomorum* BDGP5 (CP023657-CP023660) | *Drosophila melanogaster* gut | - / USA | Chromosome | Intact | 45.1 | 56101-101202 |
|  |  |  |  |  | Incomplete | 30.2 | 673405-703616 |
|  |  |  |  |  | Incomplete | 17.7 | 976729-994505 |
|  |  |  |  |  | Incomplete | 13.3 | 1563935-1577309 |
|  |  |  |  |  | Incomplete | 33.4 | 2298645-2332099 |
|  |  |  |  | Plasmid | Incomplete | 16.4 | 69243-85736 (pApBDGP5A) |
|  |  |  |  |  | Incomplete | 2.1 | 6072-8204 (pApBDGP5C) |
|  | *pomorum* DM001  (NZ_AEUP00000000.1) | *Drosophila melanogaster* gut | - / South Korea | Chromosome | Incomplete | 13.6 | 1448846-1462504 |
|  |  |  |  |  | Incomplete | 17.7 | 2269552-2287289 |
|  |  |  |  |  | Incomplete | 19.8 | 2608492-2628313 |
|  | *senegalensis* 108B  (LN606600.1) | Cocoa bean heap fermentation | - / Ghana | Chromosome | Incomplete | 32.0 | 724192-756241 |
|  |  |  |  |  | Intact | 38.8 | 852899-891751 |
|  |  |  |  |  | Intact | 45.7 | 1739190-1784945 |
|  |  |  |  |  | Questionable | 28.5 | 1899725-1928255 |
|  |  |  |  |  | Intact | 46.3 | 2319625-2366021 |
|  |  |  |  |  | Incomplete | 14.7 | 2556911-2571678 |
|  | *tropicalis* NBRC 101654 (SKU1100) | Fruits | - / Thailand | Chromosome | Incomplete | 12.9 | 1993981-2006941 |
|  | *tropicalis* BDGP1 (CP022699-CP022700) | Male feces of *Drosophila melanogaster* | - / USA | Chromosome | Intact | 37.4 | 73691-111172 |
|  |  |  |  |  | Questionable | 25.0 | 1621147-1646180 |
|  |  |  |  |  | Questionable | 23.1 | 1762032-1785155 |
|  |  |  |  |  | Questionable | 21.9 | 1795215-1817208 |
|  |  |  |  |  | Intact | 64.2 | 2859175-2923385 |
|  | sp. JWB (CP030871-CP030873) | *Drosophila melanogaster* | - / South Korea | Chromosome | Incomplete | 17.7 | 570073-587810 |
|  |  |  |  |  | Incomplete | 20.0 | 675313-695382 |
|  |  |  |  |  | Incomplete | 15.8 | 907902-923721 |
|  |  |  |  |  | Intact | 56.0 | 1491464-1547535 |
|  |  |  |  | Plasmid | Incomplete | 23.6 | 2757263-2780925 |
| *Komagataeibacter* | *europaeus* SRCM101446  (CP021467-CP021470) | Food | - / South Korea | Chromosome | Questionable | 41.5 | 1368664-1410215 |
|  |  |  |  | Plasmid | Incomplete | 11.0 | 107127-118185 (pKE1446-1) |
|  | *medellinensis* NBRC 3288  (AP012159- AP012166) | Vineger | - / - | Chromosome | Incomplete | 14.6 | 910991-925627 |
|  |  |  |  |  | Intact | 53.4 | 1729620-1783118 |
|  |  |  |  |  | Intact | 37.9 | 2353951-2391861 |
|  |  |  |  | Plasmid | Incomplete | 7.8 | 34377-42198 (pGXY010) |
|  |  |  |  |  | Questionable | 23.8 | 8744-32570 (pGXY020) |
|  |  |  |  |  | Incomplete | 11.6 | 8642-20273 (pGXY030) |
|  | *nataicola* RZS01  (CP019875-CP019881) | Rotten apples | - / China | Chromosome | Incomplete | 13.3 | 112719-126061 |
|  |  |  |  |  | Questionable | 14.6 | 1624645-1639248 |
|  |  |  |  |  | Incomplete | 7.6 | 1968636-1976276 |
|  |  |  |  |  | Intact | 36.5 | 2319672-2356227 |
|  |  |  |  |  | Intact | 12.4 | 2890299-2902793 |
|  |  |  |  |  | Intact | 24.9 | 3211434-3236357 |
|  |  |  |  | Plasmid | Incomplete | 18.5 | 5863-24367 (pKNA03) |
|  | *xylinus* E25 (CP004360-CP004365) | Vineger | Władysławowo / Poland | Chromosome | Intact | 44.4 | 224522-268964 |
|  |  |  |  |  | Incomplete | 6.2 | 802723-808975 |
|  |  |  |  |  | Incomplete | 16.4 | 2544677-2561158 |
|  |  |  |  | Plasmid | Incomplete | 13.7 | 12513-26296 (pGX3) |
|  |  |  |  |  | Incomplete | 25.7 | 157528-183244 (pGX5) |
| *Gluconacetobacter* | *albidus* TMW2.1191 (CP014689-CP014690) | Water kefir | Freising / Germany | Chromosome | Incomplete | 32.2 | 1456544-1488785 |
|  |  |  |  |  | Incomplete | 16.6 | 2845423-2862099 |
|  | *oxydans* 621H  (CP000004-CP000009) | - | - / - | Chromosome | Intact | 22.2 | 1317303-1339518 |
|  |  |  |  |  | Intact | 43.8 | 2537934-2581775 |
|  |  |  |  |  | Incomplete | 11.5 | 2674647-2686163 |
|  |  |  |  | Plasmid | Questionable | 5.4 | 82898-88321 (pGOX1) |
|  |  |  |  |  | Incomplete | 22.3 | 3107-25462 (pGOX2) |
|  |  |  |  |  | Incomplete | 13.6 | 849-14529 (pGOX3) |
|  | *oxydans* H24 (CP003926-CP003927) | - | - / - | Chromosome | Incomplete | 18.8 | 2374373-2393220 |
|  |  |  |  | Plasmid | Questionable | 10.0 | 182123-192206 (213 kbp unnamed plasmid) |
|  | *oxydans* DSM 3504 (CP004373) | - | - / - | Chromosome | Incomplete | 19.1 | 137379-156519 |

^a^ Dashes indicate unknown. ^b^ intact, incomplete, and questionable indicate active phage, degenerate phage, and phage where it was impossible to predict whether the phage is active or degenerate, respectively.
