# Supplementary material for: Distribution and genome structures of temperate phages in acetic acid bacteria
Source: Sci Rep. 2021 Nov 3;11:21567. doi: 10.1038/s41598-021-00998-w (PMC8566455; doi:10.1038/s41598-021-00998-w)
Supplement: Supplementary file 5 — Supplementary Information 5. [file 41598_2021_998_MOESM5_ESM.docx]

**Table S2 List of AAB strains used in this study**

| **Genus** | **Species** | **Culture Collection No.^a^** | **Isolation source^a^** | **Locality of Source / Country of Origin^b^** |
| --- | --- | --- | --- | --- |
| *Acetobacter* | *aceti* | NBRC 3281* | Film in fermentor of rice vinegar | Aichi / Japan |
|  |  | NBRC 14818 | Alcohol turned to vinegar | - / - |
|  |  | DSM 3508* | Alcohol turned to vinegar | - / - |
|  |  | ATCC 23747* | - | - / - |
|  |  | ATCC 23748* | - | - / - |
|  | *cerevisiae* | DSM 2324 | - | - / - |
|  | *cibinongensis* | NBRC 16605* | Fruit of mountain soursop, *Annona montanae* | Bogor / Indonesia |
|  | *estunensis* | NBRC 13751* | Cider, Bristol | - / UK |
|  |  | ATCC 23753 | - | - / - |
|  | *indonesiensis* | NBRC 16471* | Rotten zirzak (fruit) | Bogor / Indonesia |
|  | *lovaniensis* | NBRC 3248* | *Lilium auratum* | - / Japana |
|  |  | NBRC 13753* | Sewage on soil | Becquevoort / Belgium |
|  | *orientalis* | NBRC 16606* | Canna flower, *Canna hybrida* | Bogor / Indonesia |
|  | *orleanensis* | NBRC 3170* | Manufacture of vinegar | - / - |
|  |  | NBRC 3296* | Film in fermentor of rice vinegar | Aichi / Japan |
|  |  | NBRC 13752* | Beer | - / Belgium |
|  |  | ATCC 6033* | Beer | - / Netherlands |
|  |  | ATCC 6438 | Manufacture of vinegar | - / - |
|  | *pasteurianus* | NBRC 3188* | - | - / - |
|  |  | NBRC 3191* | Beer | - / Netherlands |
|  |  | NBRC 3222 | - | - / - |
|  |  | NBRC 3279 | Spoiled wine | - / Japan |
|  |  | NBRC 3280* | Spoiled wine | - / Japan |
|  |  | NBRC 3283* | Film in fermentor of rice vinegar | Aichi / Japan |
|  |  | NBRC 3284* | Vinegar | - / - |
|  |  | NBRC 3299* | Spoiled beer | Osaka / Japan |
|  |  | NBRC 109446 | Vinegar fermentor | - / Japan |
|  |  | IAM 1804 | - | - / - |
|  |  | IAM 1807 | - | - / - |
|  |  | IAM 1826 | - | - / - |
|  |  | ATCC 9322* | - | - / - |
|  |  | ATCC 9323* | - | - / - |
|  |  | ATCC 9325* | - | - / - |
|  |  | ATCC 9432* | - | - / - |
|  |  | ATCC 12873 | - | - / - |
|  |  | ATCC 12877* | - | - / - |
|  |  | ATCC 12879 | Top fermentation storage beer | - / - |
|  |  | ATCC 23650* | Turbid beer | - / - |
|  |  | ATCC 23752* | - | - / - |
|  |  | ATCC 23754* | - | - / - |
|  |  | ATCC 23757* | - | - / - |
|  |  | ATCC 23758* | - | - / - |
|  |  | ATCC 23759* | - | - / - |
|  |  | ATCC 23760* | - | - / - |
|  |  | ATCC 23761* | - | - / - |
|  |  | ATCC 23764 | - | - / - |
|  |  | ATCC 33445* | Fermented beverages | - / - |
|  |  | NCIB 8894 | Beer (ale) in storage | Toronto / Canada |
|  | *peroxydans* | ATCC 12874 | Ditch water | Delft / Netherlands |
|  | *pomorum* | DSM 11825 | Industrial cider vinegar fermentation | Southern Germany / Germany |
|  | *syzygii* | NBRC 16604* | Fruit of Malay rose apple, *Syzygium malaccense* | Bogor / Indonesia |
|  | *tropicalis* | NBRC 16470 | Coconut juice, *Coccos nucifera* | Yogyakarta / Indonesia |
|  | sp. | NBRC 3297* | Vinegar | Kobe / Japan |
|  |  | NBRC 3298* | Vinegar | Kobe / Japan |
|  |  | ATCC 8303* | - | - / - |
|  |  | ATCC 21409 | Plant-derived foodstuff | - / - |
|  |  | ATCC 21760 | - | - / - |
|  |  | ATCC 21761 | - | - / - |
|  |  | ATCC 21762 | - | - / - |
|  |  | ATCC 35002 | Fermented tea |  |
| *Komagataeibacter* | *europaeus* | NBRC 3261* | Fruit, *Myrica rubra* | Nishinomiya / Japan |
|  |  | DSM 6160 | Submerged culture vinegar generator | Esslingen / Germany |
|  | *hansenii* | NBRC 14816* | Local vinegar | Jerusalem / Israel |
|  |  | NBRC 14817 | Local vinegar | Jerusalem / Israel |
|  |  | NBRC 14820* | Local vinegar | Jerusalem / Israel |
|  |  | DSM 11804 | Tea fungus beverage (Komucha) | - / Switzerland |
|  |  | ATCC 10821 | - | - / - |
|  |  | ATCC 53582 | Sugarcane exudate | - / - |
|  | *maltaceti* | NBRC 14815* | Malt vinegar brewery acetifiers | - / - |
|  | *medellinensis* | NBRC 3288* | Vineger | - / - |
|  | *oboediens* | NBRC 14822* | - | - / - |
|  | *xylinus* | NBRC 13693 | - | - / - |
|  |  | NBRC 13772 | Film in fermentor of vinegar | - / - |
|  |  | NBRC 13773 | Film in fermentor of vinegar | - / - |
|  |  | NBRC 15237 | Mountains ash berries | - / - |
|  |  | ATCC 10245* | - | - / - |
|  |  | ATCC 12878* | Vinegar | - / - |
|  |  | ATCC 14851 | - | - / - |
|  |  | ATCC 23766 | - | - / - |
|  |  | ATCC 31174 | - | - / - |
|  |  | ATCC 53263* | - | - / - |
|  |  | ATCC 53264* | - | - / - |
|  |  | ATCC 53524 | - | - / - |
|  |  | ATCC 53749 | - | - / - |
|  |  | ATCC 53750 | - | - / - |
| *Gluconacetobacter* | *diazotrophicus* | ATCC 49037* | Sugarcane roots | Alagoas / Brazil |
|  |  | ATCC 49038* | Sugarcane | Parnambuco / Brazil |
|  | *frateurii* | NBRC 3251 | Flower, *Dahlia* sp. | Kyoto / Japan |
|  | *liquefaciens* | NBRC 12388* | Dried persimmon, *Diospyros* sp. | - / Japan |
|  |  | IAM 1835 | Fruit | - / Japan |
|  |  | ATCC 23751 | - | - / - |
|  | *oxydans* | NBRC 3244 | - | - / - |
|  | *sacchari* | DSM 12717 | Mealy bug from sugar cane | Queensland / Australia |
| *Gluconobacter* | *albidus* | NBRC 3250 | *Dahlia coccinea* | - / - |
|  |  | NBRC 3273 | Fruit, *Myrica rubra* | Nishinomiya / Japan |
|  | *cerinus* | NBRC 3267 | Cherry, *Prunus* sp. | Osaka / Japan |
|  |  | NBRC 3274 | Fruit, *Myrica rubra* | Nishinomiya / Japan |
|  |  | NBRC 3275* | Flower, *Rheum undulatum* | Kyoto / Japan |
|  |  | NBRC 3276 | Flower, *Rheum undulatum* | Kyoto / Japan |
|  |  | IAM 1832 | - | - / - |
|  |  | ATCC 15179 | - | - / - |
|  |  | ATCC 23775 | - | - / - |
|  |  | ATCC 23777* | - | - / - |
|  |  | ATCC 43781 | Pineapple | Hawaii / USA |
|  | *frateurii* | NBRC 3253 | Flower, *Dahlia* sp. | Kyoto / Japan |
|  |  | NBRC 3262 | Strawberry, *Fragaria ananassa* | Osaka / Japan |
|  |  | NBRC 3264 | Strawberry, *Fragaria ananassa* | Osaka / Japan |
|  |  | NBRC 3265 | Strawberry, *Fragaria ananassa* | Osaka / Japan |
|  |  | NBRC 3268 | Cherry, *Prunus* sp. | Osaka / Japan |
|  |  | NBRC 3270* | Cherry, *Prunus* sp. | Osaka / Japan |
|  |  | NBRC 3285 | Fruit, *Eriobotrya japonica* | Osaka / Japan |
|  |  | NBRC 3286 | Fruit, *Eriobotrya japonica* | Osaka / Japan |
|  |  | NBRC 3290 | Persimmons, *Diospyros* sp. | Osaka / Japan |
|  |  | ATCC 9324 | - | - / - |
|  |  | ATCC 15178 | - | - / - |
|  |  | IAM 1815 | - | - / - |
|  | *japonicus* | NBRC 3260 | Fruit, *Myrica rubra* | Nishinomiya / Japan |
|  |  | NBRC 3263* | Strawberry, *Fragaria ananassa* | Osaka / Japan |
|  |  | NBRC 3269 | Cherry, *Prunus* sp. | Osaka / Japan |
|  |  | NBRC 3271 | Fruit, *Myrica rubra* | Nishinomiya / Japan |
|  |  | NBRC 3272* | Fruit, *Myrica rubra* | Nishinomiya / Japan |
|  |  | ATCC 15180 | - | - / - |
|  | *kondonii* | NBRC 3266 | Strawberry, *Fragaria ananassa* | Osaka / Japan |
|  | *oxydans* | NBRC 3130 | - | - / - |
|  |  | NBRC 3189 | Vinegar | - / - |
|  |  | NBRC 3287* | Flower, *Liatris scariosa* | Kyoto / Japan |
|  |  | NBRC 3292 | - | - / - |
|  |  | NBRC 3294 | Dried persimmons, *Diospyros* sp. | Osaka / Japan |
|  |  | NBRC 3462* | - | - / - |
|  |  | NBRC 12528 | - | - / - |
|  |  | NBRC 14819 | Beer | - / - |
|  |  | IAM 1813 | - | - / - |
|  |  | IAM 1838 | Probably *Diospyros* sp. | Tokyo / Japan |
|  |  | IAM 1839 | - | - / - |
|  |  | IAM 12138 | - | - / - |
|  |  | ATCC 621H | - | - / - |
|  |  | ATCC 8147 | - | - / - |
|  |  | ATCC 9433* | Vinegar | - / - |
|  |  | ATCC 9844 | - | - / - |
|  |  | ATCC 9937 | - | - / - |
|  |  | ATCC 11894 | - | - / - |
|  |  | ATCC 11895 | - | - / - |
|  |  | ATCC 15163 | - | - / - |
|  |  | ATCC 19357* | Fermented beverages, beer | - / - |
|  |  | ATCC 23651 | Cider | - / - |
|  |  | ATCC 23652 | - | - / - |
|  |  | ATCC 23755 | - | - / - |
|  |  | ATCC 23771 | - | - / - |
|  |  | ATCC 23772 | Beer | - / - |
|  |  | ATCC 23776 | - | - / - |
|  |  | ATCC 33447* | Fermented beverages |  |
|  |  | ATCC 33448 | Amstel beer | Delft / Netherlands |
|  | *sphaericus* | NBRC 12467* | Grape | - / Japan |
|  | *thailandicus* | NBRC 3172 | - | - / - |
|  |  | NBRC 3225 |  | - / - |
|  |  | NBRC 3254 | Strawberry, *Fragaria chilonensis var. ananassa* | Osaka / Japan |
|  |  | NBRC 3255 | Strawberry, *Fragaria chilonensis var. ananassa* | Osaka / Japan |
|  |  | NBRC 3257 | Fruit, *Prunus tomentosa* | Osaka / Japan |
|  |  | NBRC 3258* | Fruit, *Prunus tomentosa* | Osaka / Japan |
|  |  | NBRC 3289 | Persimmons, *Diospyros* sp. | Osaka / Japan |
|  |  | NBRC 3291 | Persimmons, *Diospyros* sp. | Osaka / Japan |
|  |  | NBRC 3259 | Flower of dahlia | - / - |
|  | sp. | IAM 1827* | - | - / - |
|  |  | IAM 12139 | - | - / - |
|  |  | ATCC 15164 | - | - / - |
| *Acidomonas* | *methanolica* | JCM 6891 | Methanol fermentation with *Candida* sp. (not sterile) | Leipzig / Germany |
|  |  | ATCC 43582 | Ditch sludge | East Germany / Germany |
| *Ameyamaea* | *chiangmaiensis* | NBRC 103196 | Flower of red ginger, *Alpinia purpurea* | Chaing Mai / Thailand |
|  |  | NBRC 16594 | Flower of orchid, *Bauhinia purpurea* | Bogor / Indonesia |
| *Asaia* | *bogorensis* | NRIC 311 | - | - / - |
|  | *siamensis* | NBRC 16457 | Flower of crown flower, *Calotropis gigantea* | Bangkok / Thailand |
|  |  | NRIC 323 | - | - / - |
| *Frateuria* | *aurantia* | NBRC 13332 | Fruit of raspberry, *Rubus cuneifolius* | - / Japan |
| *Kozakia* | *baliensis* | NRIC 485 | - | - / - |
|  |  | NRIC 487 | - | - / - |
|  |  | NRIC 488 | - | - / - |
| *Neoasaia* | *chiangmaiensis* | NBRC 101099 | Flower, Alpinia purpurata | Chaing-Mai / Thailand |

^a^ Asterisks indicate the bacteria tested as a host for p2096int vector. ^b^ Dashes indicate unknown.
