# Supplementary material for: Distribution and genome structures of temperate phages in acetic acid bacteria
Source: Sci Rep. 2021 Nov 3;11:21567. doi: 10.1038/s41598-021-00998-w (PMC8566455; doi:10.1038/s41598-021-00998-w)
Supplement: Supplementary file 6 — Supplementary Information 6. [file 41598_2021_998_MOESM6_ESM.docx]

**Table S3 MMC-sensitive AAB strains**

| **Genus** | **Species** | **Culture collection No.** | **MIC of MMC (μg/mL)** |
| --- | --- | --- | --- |
| *Acetobacter* | *estunensis* | NBRC 13751 | 4.0 |
|  | *orleanensis* | NBRC 3170 | 0.2 |
|  |  | NBRC 3296 | 1.0 |
|  |  | ATCC 6438 | 0.2 |
|  | *pasteurianus* | NBRC 3283 | 4.0 |
|  |  | NBRC 109446 | 4.0 |
|  |  | ATCC 9432 | 1.0 |
|  | sp. | ATCC 21409 | 0.2 |
|  |  | ATCC 21760 | 0.2 |
|  |  | ATCC 35002 | 4.0 |
| *Komagataeibacter* | *hansenii* | ATCC 10821 | 2.0 |
|  | *maltaceti* | NBRC 14815 | 4.0 |
|  | *xylinus* | NBRC 13693 | 4.0 |
|  |  | NBRC 13772 | 4.0 |
|  |  | NBRC 13773 | 4.0 |
|  |  | ATCC 14851 | 4.0 |
|  |  | ATCC 53264 | 4.0 |
| *Gluconacetobacter* | *diazotrophicus* | ATCC 49037 | 4.0 |
| *Gluconobacter* | *albidus* | NBRC 3273 | 2.0 |
|  | *cerinus* | IAM 1832 | 0.2 |
|  |  | ATCC 23755 | 4.0 |
|  | *oxydans* | IAM 1838 | 1.0 |
|  |  | ATCC 9324 | 4.0 |
|  |  | ATCC 9433 | 2.0 |
| *Acidomonas* | *methanolica* | ATCC 43582 | 0.2 |
| *Ameyamaea* | *chiangmaiensis* | NBRC 103196 | 2.0 |
| *Frateuria* | *aurantia* | NBRC 13332 | 0.2 |
