# Supplementary material for: Distribution and genome structures of temperate phages in acetic acid bacteria
Source: Sci Rep. 2021 Nov 3;11:21567. doi: 10.1038/s41598-021-00998-w (PMC8566455; doi:10.1038/s41598-021-00998-w)
Supplement: Supplementary file 7 — Supplementary Information 7. [file 41598_2021_998_MOESM7_ESM.docx]

**Table S5. Oligonucleotide primers used in study**

| **Name** | **Sequence (5′-3′) *^a^*** | **Restriction enzyme *^b^*** |
| --- | --- | --- |
| M30-ck3F | GATTATCCAGAGGGGTATAAGTTTTTC | - |
| M30-ck3R | GATGTTCTGTTGTTTCGGCTTC | - |
| M30-ck1F | AATACGTTCACCCGATTCAATAAA | - |
| M30-ck1R | CTTCTGCCCTTCTAGTATGAGTCTG | - |
| M30-ck2F | ACATGACAAAATCTTGCTTATCAGATG | - |
| M30-ck2R | GTATGGCTCGTATAATCTAAATTGCAG | - |
| Dis19188F | GAATTCTCCATAGGGTTTGCTTC | *Eco*RI |
| Dis20547R | GGATCCTTGTTTTTCTGGATCG | *Bam*HI |
| 2096int-F1 | CATATGTCGCATTGAGAAGGAAGTAA | *Nde*I |
| 2096int-R1 | TCGCTCATACGCTGGCGGATGCGATGCGCTGTTTCCACCG | - |
| 2096int-F2 | CGGTGGAAACAGCGCATCGCATCCGCCAGCGTATGAGCGA | - |
| 2096int-R2 | CATATGGTCAGCCTATTCACGGCACA | *Nde*I |
| 2096int-M-R | TTCCCTATAGTTCCCAATTATTC | - |
| 2096int-M1-L | GTTGGCGGATGGGGTGTCAGT | - |
| 2096int-M2-L | TGGCGGATGGGGTGTCAGTC | - |
| 2096int-M3-L | GGCGGATGGGGTGTCAGTCAG | - |
| tRNASer_attB_F2 | AACCCACCATCTGGGTATTG | - |
| tRNASer_attB_R4 | AACCGTCTTCCGTCCGAACC | - |

*^a^* Restriction sites are underlined. *^b^* Dashes indicate the absence of restriction site.
